# Comparative efficacy of dietary interventions for glycemic control and pregnancy outcomes in gestational diabetes: a network meta-analysis of randomized controlled trials

**DOI:** 10.3389/fendo.2025.1512493

**Published:** 2025-05-19

**Authors:** Jiaoyang Di, Jingjing Fan, Fangxu Ma

**Affiliations:** ^1^ Department of Obstetrics and Gynecology, Hebei General Hospital, Shijiazhuang, China; ^2^ Department of Respiratory and Critical Care Medicine, Hebei Chest Hospital, Shijiazhuang, China

**Keywords:** gestational diabetes mellitus, dietary interventions, network meta-analysis, glycemic control, adverse pregnancy outcomes

## Abstract

**Background:**

Gestational diabetes mellitus (GDM) poses significant risks to both maternal and fetal health, and effective dietary interventions are critical for managing the condition. This study aimed to evaluate the efficacy of various dietary interventions on glycemic control and adverse pregnancy outcomes in GDM patients through a network meta-analysis.

**Methods:**

A systematic review and network meta-analysis of randomized controlled trials (RCTs) were conducted in accordance with PRISMA guidelines. Data were sourced from PubMed, MEDLINE, Embase, Web of Science, and CNKI up to September 3, 2024. The primary outcomes were fasting blood glucose (FBG), 2-hour postprandial blood glucose (2h-PBG), insulin resistance (HOMA-IR), and adverse pregnancy outcomes, including cesarean section, macrosomia, and gestational hypertension. Effect sizes were reported as odds ratios (OR) for dichotomous outcomes and mean differences (MD) or standardized mean differences (SMD) for continuous outcomes, with 95% confidence intervals (CI).

**Results:**

A total of 28 RCTs with 2666 participants were included, evaluating seven distinct dietary interventions. Among them, 19 studies assessed the low-glycemic index (Low-GI) diet, 4 evaluated the Dietary Approaches to Stop Hypertension (DASH) diet, 4 investigated low-carbohydrate diets, 1 examined the low-glycemic load (Low-GL) diet, and 1 explored a combined low-carbohydrate and DASH diet. The remaining trials compared standard dietary recommendations or structured meal planning. The DASH diet was the most effective intervention for glycemic control, significantly reducing FBG (SMD = -2.35, 95% CI [-4.15, -0.54]), 2h-PBG (SMD = -1.41, 95% CI [-2.56, -0.25]), and HOMA-IR (MD = -1.90, 95% CI [-2.44, -1.36]). Both the DASH and Low-GI diets significantly reduced adverse pregnancy outcomes. Specifically, the DASH diet significantly reduced the risk of cesarean section (OR = 0.54, 95% CI [0.40, 0.74]), while the Low-GI diet significantly reduced the risk of macrosomia (OR = 0.12, 95% CI [0.03, 0.51]).

**Conclusion:**

This network meta-analysis suggests that the DASH and Low-GI diets may be beneficial for managing gestational diabetes mellitus. The DASH diet showed favorable trends in improving glycemic control, while both diets appeared to reduce the risks of cesarean delivery and macrosomia. Further high-quality research is needed to confirm these findings and optimize dietary recommendations for clinical practice.

**Systematic review registration:**

https://www.crd.york.ac.uk/PROSPERO/, identifier CRD420251008181.

## 1 Introduction

Gestational diabetes mellitus (GDM) is defined as glucose intolerance that is first recognized during pregnancy, typically in the second or third trimester, in women without a prior history of diabetes (1). This condition is a major public health concern due to its significant impact on both maternal and fetal health. GDM increases the risk of short- and long-term complications for both the mother and the offspring. In the short term, GDM is associated with fetal complications, including excessive fetal growth, which may lead to macrosomia, as well as an increased risk of preterm birth and neonatal hypoglycemia (2, 3). In the long term, women with GDM are at a higher risk of developing type 2 diabetes mellitus (T2DM), and their offspring are more likely to experience metabolic disorders and obesity later in life (4). Moreover, the rising incidence of GDM has placed substantial economic burdens on families, individuals, and healthcare systems globally (5). The prevalence of GDM has been increasing worldwide, reflecting the rising rates of obesity and sedentary lifestyles. Current estimates indicate that GDM affects approximately 10-15% of pregnancies globally, with significant regional variations (6, 7). In countries such as China, the prevalence of GDM has surged in recent years, making it a critical focus of public health interventions (8).

Dietary interventions play a crucial role in managing GDM. Encouraging the adoption of a healthy diet helps to maintain optimal glucose metabolism, supports appropriate gestational weight gain, and meets the nutritional needs of the growing fetus, all while reducing the risk of adverse complications (9). The National Institute for Health and Care Excellence (NICE) guidelines highlight that structured dietary planning can significantly improve pregnancy outcomes compared to no dietary intervention (10). For women with GDM, dietary patterns should include sufficient micronutrients to sustain fetal growth while limiting postprandial glucose spikes (11). These interventions also promote controlled maternal weight gain, reducing the likelihood of complications such as macrosomia and preterm birth. The occurrence of both hyperglycemia and hypoglycemia is directly influenced by carbohydrate intake, underscoring the importance of carbohydrate management in dietary interventions (12). Thus, modifying dietary patterns is critical to effectively managing GDM and mitigating the risks associated with the condition.

Currently, common dietary interventions for managing GDM both domestically and internationally include low-glycemic index (Low-GI) diets, the Dietary Approaches to Stop Hypertension (DASH) diet, low-carbohydrate diets, and low-glycemic load (Low-GL) diets (13). Studies have demonstrated the benefits of these dietary approaches in improving glycemic control and reducing pregnancy-related complications. For example, Low-GI diets have been associated with better postprandial glucose regulation, while the DASH diet has shown promise in reducing blood pressure and improving metabolic outcomes in GDM patients (14). Low-carbohydrate diets, on the other hand, help limit glucose spikes by reducing carbohydrate intake (15).

The selection of these dietary interventions was based on their frequent evaluation in RCTs specific to GDM. These diets have been widely studied for their impact on glycemic control, insulin resistance, and adverse pregnancy outcomes, making them the most relevant for this network meta-analysis. We also included combinations of dietary patterns, such as the Low-carb DASH diet, due to potential synergistic benefits in GDM management. A recent network meta-analysis evaluated various lifestyle interventions, including dietary modifications and resistance exercise, in GDM management (16). While this study also identified the DASH and Low-GI diets as beneficial, our research focuses exclusively on dietary interventions. By using an NMA approach, we provide a comparative ranking of dietary patterns, allowing for a more targeted analysis of nutritional strategies specific to GDM. Although the Mediterranean diet has shown benefits in general metabolic health, there is currently limited RCT-based evidence directly assessing its effectiveness in GDM management relative to other dietary interventions included in this analysis (17). Given our study’s focus on synthesizing robust evidence from RCTs in GDM populations, we prioritized dietary patterns that have been extensively studied within this specific context.

A network meta-analysis offers a powerful approach to overcome this limitation. By quantitatively analyzing the effects of multiple interventions within the same set of participants, a network meta-analysis allows for indirect comparisons across different dietary patterns (18). This method enables the identification of the most effective dietary intervention for GDM management, providing more precise evidence to inform clinical decision-making. This study employs a network meta-analysis of RCTs to compare the effectiveness of different dietary interventions for GDM. It provides a comprehensive assessment of the impact of various dietary patterns on glycemic control, insulin resistance, and adverse neonatal outcomes in GDM patients. The results will offer critical insights into optimal dietary strategies for GDM management, guiding healthcare providers in selecting evidence-based nutritional interventions to improve maternal and fetal health outcomes.

## 2 Methods

This systematic review and network meta-analysis were prospectively registered in PROSPERO (Registration Number: CRD420251008181) and conducted in accordance with the Preferred Reporting Items for Systematic Reviews and Meta-Analyses (PRISMA) guidelines (19).

### 2.1 Search strategy

A comprehensive literature search was performed across multiple databases, including PubMed, MEDLINE, Embase, Web of Science, and the China National Knowledge Infrastructure (CNKI), up to September 3, 2024, without language restrictions. Both Medical Subject Headings (MeSH) terms and free-text keywords were used in the search. The search terms included: “low-carbohydrate diet,” “low-glycemic index (Low-GI) diet,” “low-glycemic load (Low-GL) diet,” “dietary intervention,” “nutritional therapy,” and “gestational diabetes mellitus,” along with keywords related to glycemic control and pregnancy outcomes. The detailed search strategy, including specific terms and dates, is provided in **Supplementary File 1**. Additionally, reference lists of relevant articles and reviews were manually searched to identify further studies. Two independent reviewers screened the titles, abstracts, and full texts of the identified studies. Any disagreements were resolved through discussion or consultation with a third reviewer, if necessary.

### 2.2 Inclusion and exclusion criteria

The inclusion criteria for this systematic review and network meta-analysis were as follows: a) Studies involving participants with a confirmed diagnosis of GDM, as defined by a 75 g oral glucose tolerance test (OGTT) with one or more abnormal values: fasting glucose >5.1 mmol/L (92 mg/dL), 1-hour post-load glucose >10.0 mmol/L (180 mg/dL), or 2-hour post-load glucose >8.5 mmol/L (153 mg/dL); b) Participants with similar baseline characteristics, including maternal age (18-45 years), parity (primiparous or multiparous), pre-pregnancy BMI (18.5-35.0 kg/m^2^), and gestational age at GDM diagnosis (typically between 24 and 32 weeks of gestation). Studies were included if they either reported comparable distributions of these variables or conducted adjustments to account for baseline differences; c) The intervention included one of the seven specified dietary patterns, either alone or in combination (low-glycemic index (Low-GI) diet, Low-GI diet with standard care, Dietary Approaches to Stop Hypertension (DASH) diet, low-glycemic load (Low-GL) diet, low-carbohydrate (Low-carb) diet, low-carbohydrate diet with DASH (Low-carb DASH), and standard care); d) Only randomized controlled trials (RCTs) were included to ensure the highest level of evidence for comparative efficacy; e) Studies published in English or Chinese were considered for inclusion.

Exclusion criteria included: a) Studies involving participants with other endocrine disorders (e.g., pre-existing diabetes, thyroid dysfunction, or polycystic ovary syndrome) to ensure the specificity of GDM-related findings; b) Interventions combining dietary approaches with other lifestyle modifications (e.g., increased physical activity, pharmacological interventions) unless dietary effects could be isolated; c) Studies with incomplete or missing data, defined as studies where essential outcome measures (e.g., fasting blood glucose, 2-hour postprandial blood glucose, insulin resistance, or pregnancy outcomes) were unavailable, inconsistently reported, or could not be retrieved from corresponding authors despite reasonable attempts to obtain the data; d) Studies with major data errors, including statistical inconsistencies between text and tables, implausible numerical values, or conflicting versions of the same dataset without adequate justification. Studies flagged for potential data fabrication or with serious methodological inconsistencies affecting data reliability were also excluded; e) Studies where the full text was unavailable, preventing quality assessment and data extraction.

### 2.3 Data extraction

After identifying relevant studies from the specified databases, EndNote X9 was used for systematic reference management. Two independent reviewers extracted data from studies that met the inclusion criteria. Discrepancies were resolved by consensus among all authors. Extracted data included publication details (author names, year, and journal), demographic characteristics of participants (age, gender), baseline glucose levels, intervention and control group details, and study outcomes. For studies lacking essential data, authors were contacted up to four times over a six-week period to obtain the necessary information.

### 2.4 Outcomes

The primary outcomes assessed were: a) Fasting Blood Glucose Levels (FBG): FBG reflects baseline glycemic control after an overnight fast and is a key marker for diagnosing and managing gestational diabetes mellitus (GDM). b) 2-Hour Postprandial Blood Glucose Levels (2h-PBG): This measures blood glucose levels two hours after a meal and is critical for evaluating postprandial glucose regulation in GDM patients. c) Insulin Resistance Index (HOMA-IR): HOMA-IR quantifies insulin resistance, which is commonly elevated in GDM. d) Incidence of Adverse Pregnancy Outcomes: This includes complications such as preeclampsia, preterm birth, macrosomia, and neonatal hypoglycemia, which are associated with poorly controlled GDM. To ensure consistency across studies, standardized definitions were applied: macrosomia was defined as birth weight ≥4000 g, preterm birth as delivery before 37 weeks of gestation, preeclampsia as blood pressure ≥140/90 mmHg with proteinuria ≥300 mg in a 24-hour urine sample, and neonatal hypoglycemia as plasma glucose <2.6 mmol/L (47 mg/dL) within the first 48 hours postpartum (20, 21). Each outcome provides key insights into the efficacy of dietary interventions in GDM management.

### 2.5 Risk of bias assessment

The risk of bias in the included studies was assessed at the study level using the revised Cochrane risk-of-bias tool (RoB 2) (22). This assessment covered the following domains: the randomization process, deviations from intended interventions, missing outcome data, measurement of outcomes, and selection of the reported result. Any disagreements in the assessments were resolved through consultation with a third reviewer, ensuring a rigorous and unbiased evaluation of the included studies.

In addition, the certainty of evidence for each outcome was assessed using the Grading of Recommendations, Assessment, Development, and Evaluations (GRADE) approach. The evaluation considered study limitations (risk of bias), inconsistency (heterogeneity), indirectness, imprecision, and publication bias. Based on these criteria, we classified the certainty of evidence for each outcome as high, moderate, low, or very low.

### 2.6 Data synthesis

**Figure 1:**
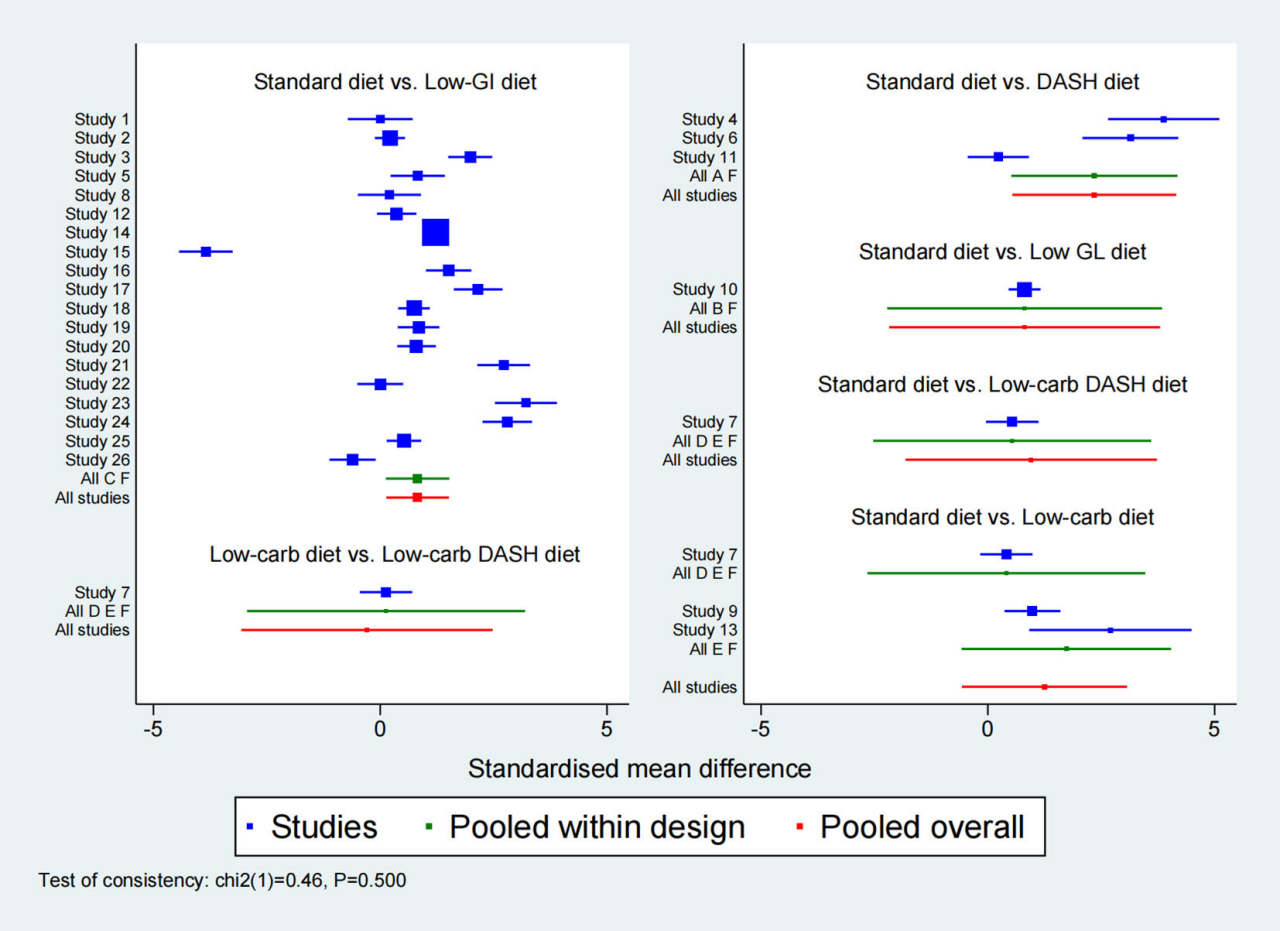
Forest plot of fasting blood glucose (FBG).

**Figure 2:**
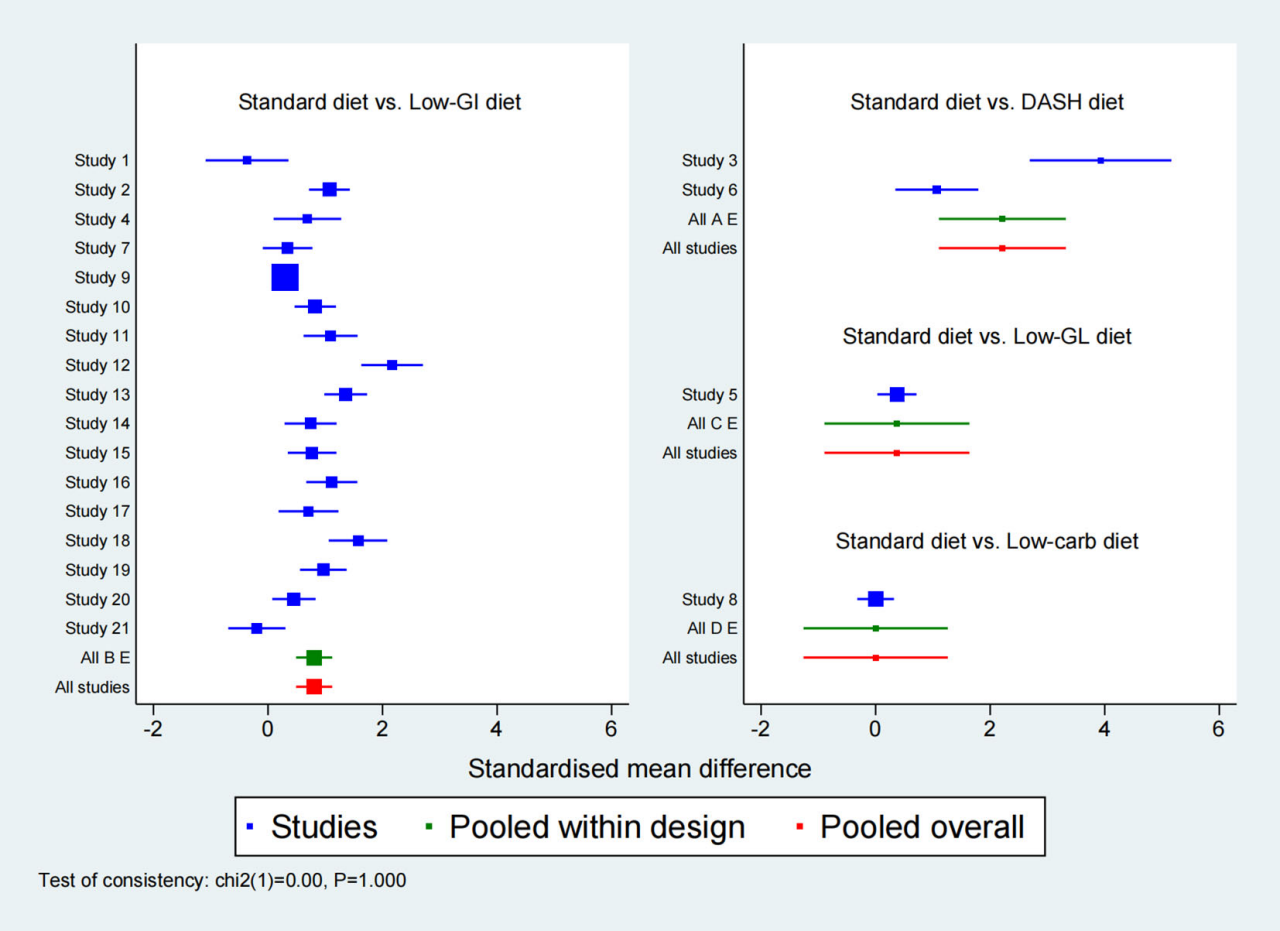
Forest plot of 2-hour postprandial blood glucose level (2h-PBG).

**Figure 3:**
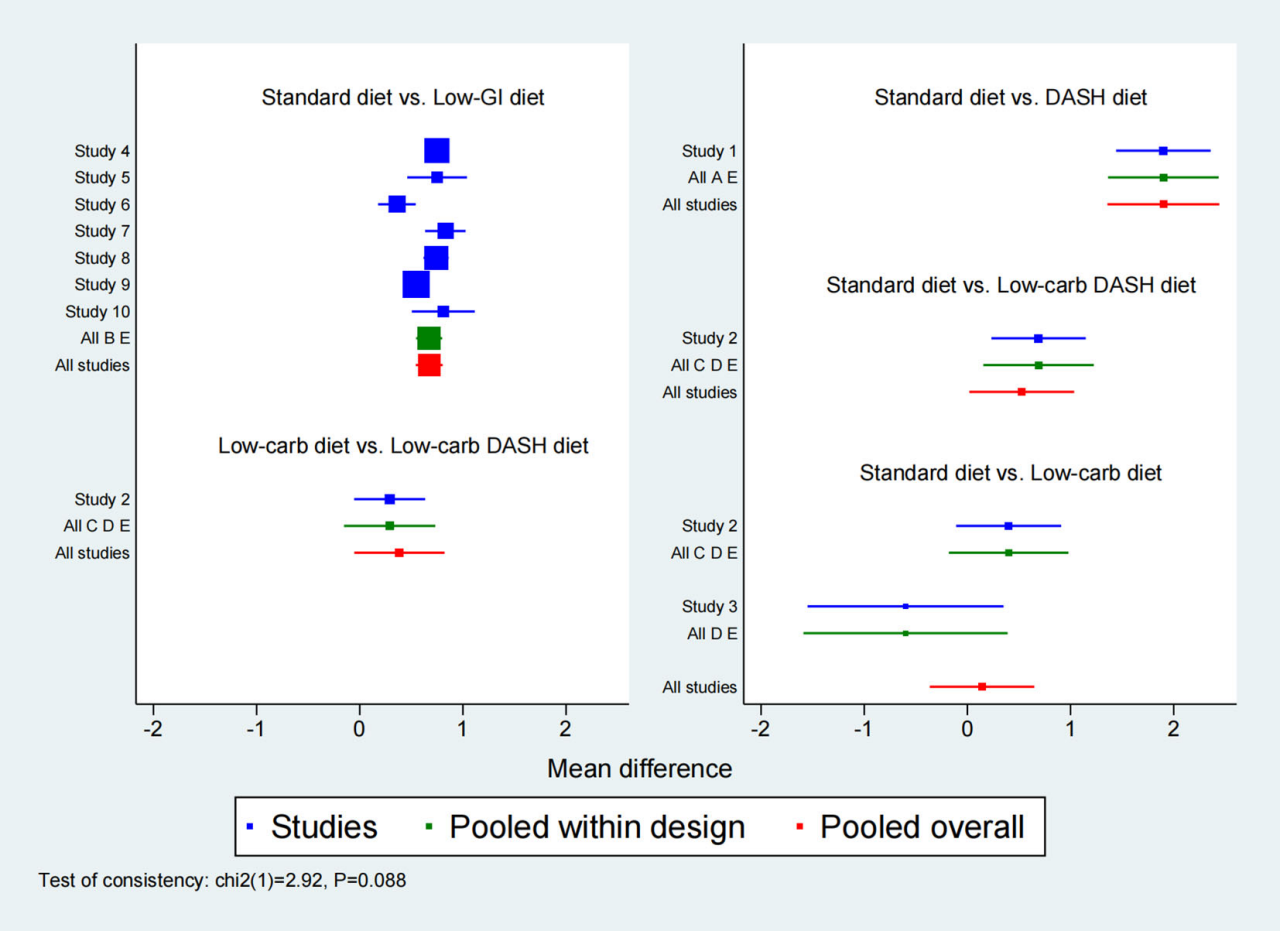
Forest plot of Insulin resistance index (HOMA-IR).

**Figure 4:**
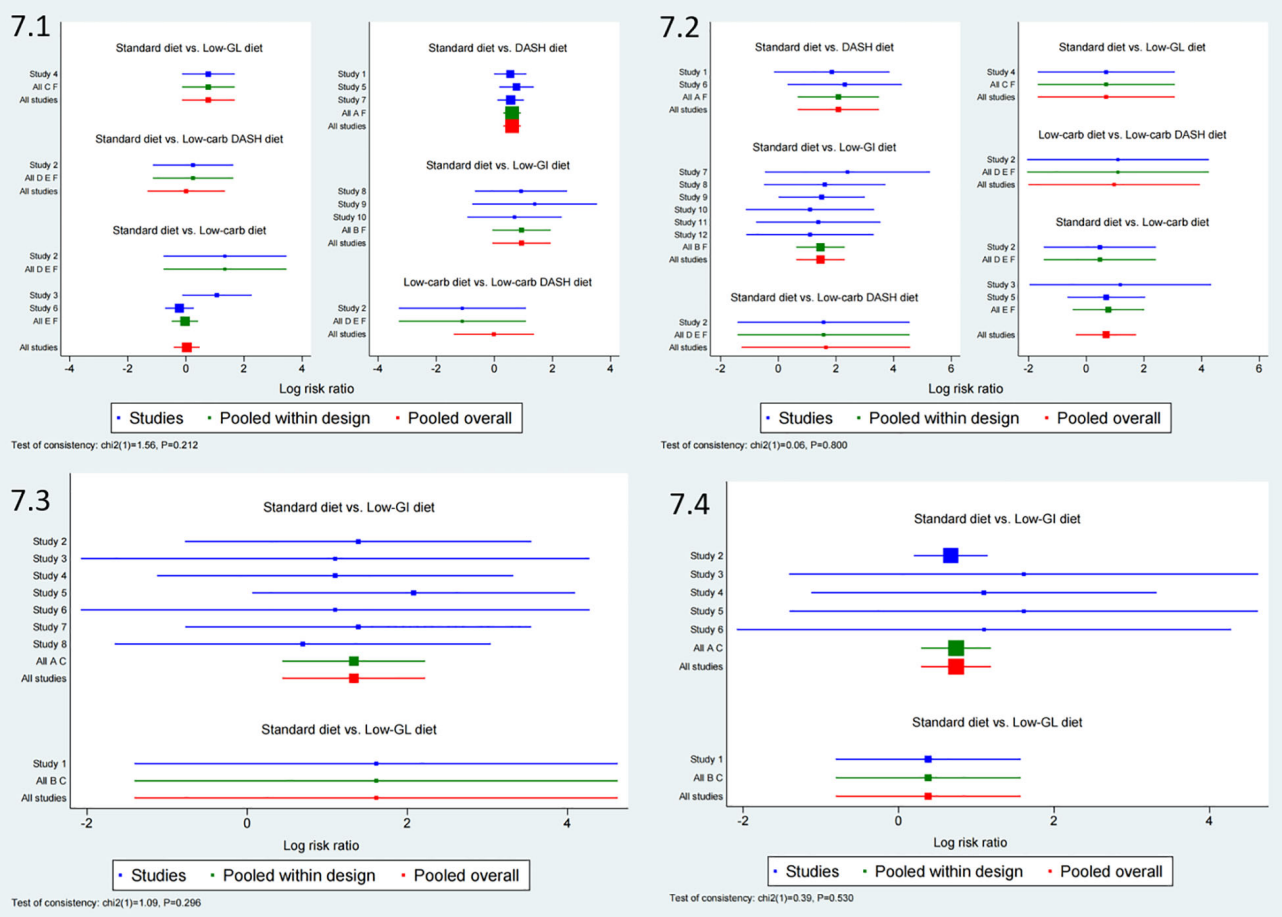
Forest plot of adverse pregnancy outcomes. 1: Cesarean Section, 2: Macrosomia, 3: Gestational Hypertension, 4: Preterm Birth.

Data synthesis was performed using Stata software (Version 17.0, StataCorp LLC, Texas, USA). A network plot was generated to visualize the comparison network and ensure the feasibility of the network meta-analysis. Bayesian network meta-analysis was conducted using the “network” and “mvmeta” packages in Stata to compare the effects of different dietary patterns on GDM outcomes. Relative risks (RRs) were used to assess dichotomous outcomes, such as the incidence of adverse pregnancy outcomes, while mean differences (MDs) were used for continuous outcomes. For outcomes measured using different units, such as fasting and 2-hour postprandial blood glucose, standardized mean differences (SMDs) were applied to assess effect sizes. To ensure a more comprehensive presentation of the meta-analysis results, forest plots for key outcomes (FBG, 2h-PBG, HOMA-IR, cesarean section, macrosomia, gestational hypertension, and preterm birth) have been included in **Figures 1**–**4**. The credibility of the estimates was evaluated using 95% confidence intervals (CI). A random-effects model was employed to account for potential heterogeneity. Surface under the cumulative ranking (SUCRA) probabilities were used to rank the treatments. Funnel plots were utilized to assess potential publication bias, and between-study heterogeneity was evaluated using statistical methods (23). Egger’s test was applied to detect publication bias, with P-values <0.05 considered indicative of potential bias.

## 3 Results

**Figure 5:**
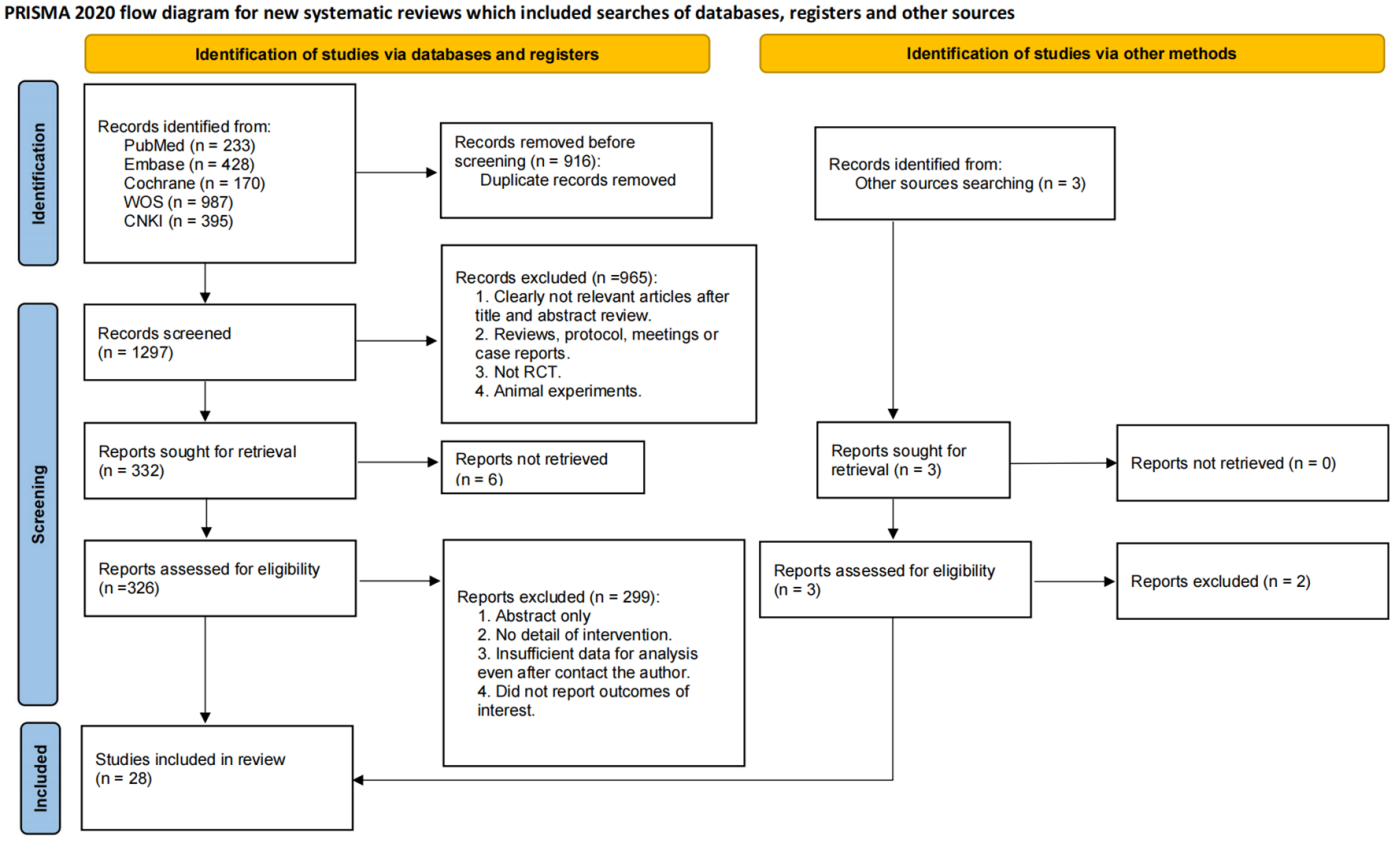
PRISMA Flow diagram of the search process for studies.

The initial electronic search yielded 2213 records. After removing duplicates, 916 records were screened based on titles and abstracts. Following the full-text review of 326 articles, a total of 28 studies, with 2666 GDM patients, met the inclusion criteria and were included in the systematic review (24–51). The PRISMA flow diagram illustrating the screening process is presented in **Figure 5**.

### 3.1 Characteristics of included studies

The 28 included studies were published between 2007 and 2024, with a median publication year of 2019. The sample sizes ranged from 12 to 566 participants, with a median of 80 participants. The age of GDM patients was reported in 26 studies, with mean ages ranging from 26.39 to 35 years, and a median age of 30.26 years. BMI was reported in 16 studies, with mean BMI values ranging from 21.2 kg/m² to 33.4 kg/m², and a median BMI of 25.9 kg/m².

Of the 28 studies, 19 employed a low-glycemic index (Low GI) diet in the intervention group, 4 utilized a DASH diet, 4 used a low-carbohydrate diet, 1 employed a low-glycemic load (Low GL) diet, and 1 used a combination of the low-carbohydrate and DASH diets. All control groups followed a standard diet regimen. In terms of outcomes, 26 studies measured FBG levels pre- and post-intervention, 21 studies reported on 2-hour 2h-PBG levels, and 10 studies assessed changes in insulin resistance. Additionally, 10 studies examined the incidence of cesarean section, 12 studies analyzed the occurrence of macrosomia, 8 studies evaluated gestational hypertension, and 6 studies investigated preterm birth rates.

### 3.2 The results of network meta-analysis

#### 3.2.1 FBG

In the network meta-analysis of FBG, 26 studies were included to evaluate the effects of different dietary interventions on FBG levels in GDM patients. **Figure 6.1** presents the direct comparisons between dietary interventions and the distribution of sample sizes. As shown in **Supplementary Table 5.1**, both the DASH diet (SMD = -2.35, 95% CI: -4.15 to -0.54) and Low-GI diet (SMD = -0.82, 95% CI: -1.52 to -0.13) significantly reduced FBG compared to the Standard diet. In terms of SUCRA rankings for FBG reduction (**Figure 7.1**), the top three interventions were DASH diet (86.8%), Low-carb diet (58.7%), and Low-carb DASH diet (49.1%), with the Standard diet having the lowest score of 13.0%. The corresponding forest plot (**Figure 1**) provides a detailed visualization of the study-level estimates and pooled effects.

**Figure 6:**
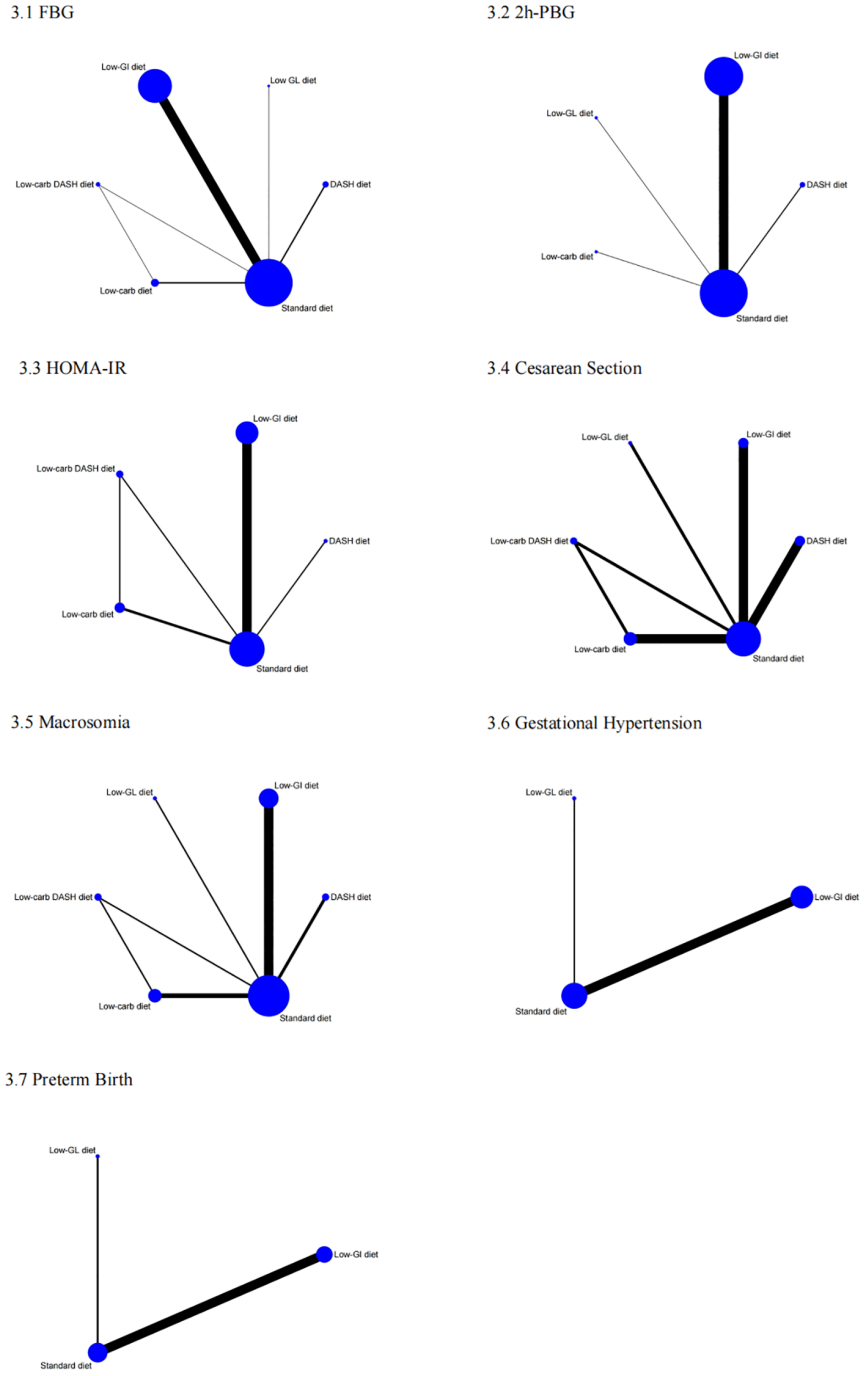
Network of eligible treatment comparisons for 1: FBG, 2: 2h-PBG, 3: HOMA-IR, 4: Cesarean Section, 5: Macrosomia, 6: Gestational Hypertension, 7: Preterm Birth.

**Figure 7:**
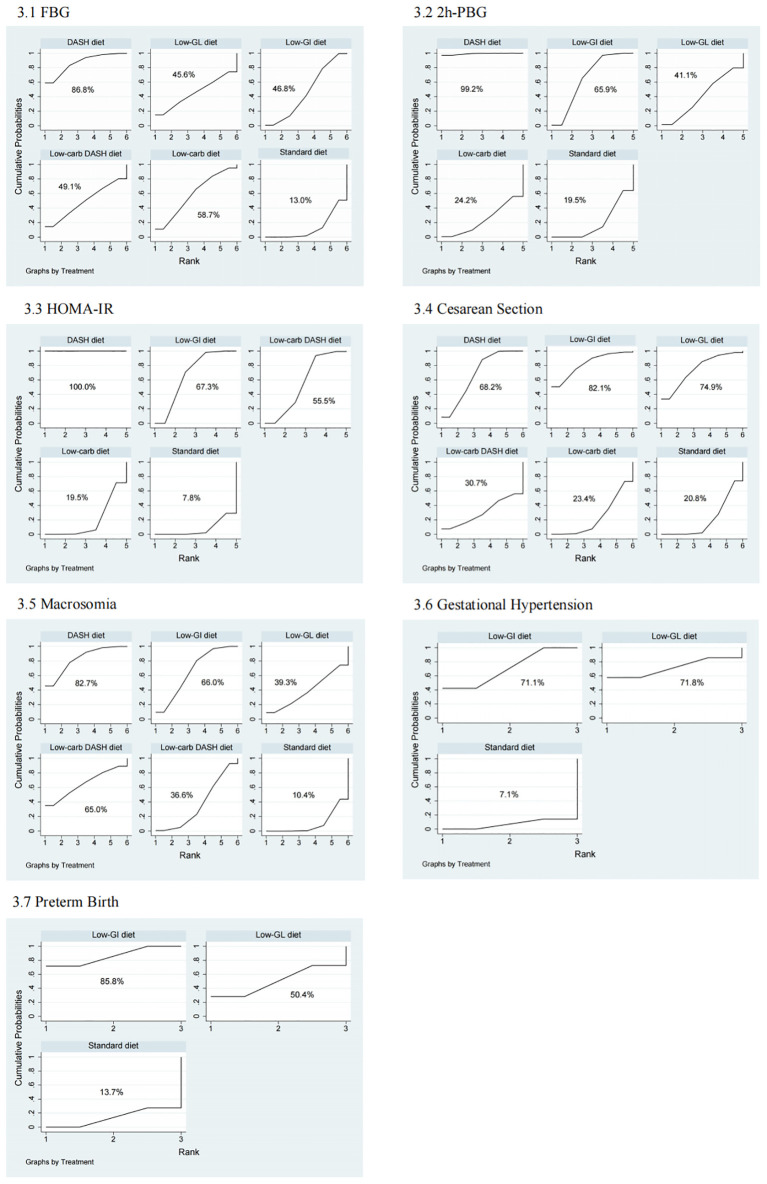
Ranking of treatment strategies based on probability of their effects for 1: FBG, 2: 2h-PBG, 3: HOMA-IR, 4: Cesarean Section, 5: Macrosomia, 6: Gestational Hypertension, 7: Preterm Birth.

#### 3.2.2 2h-PBG

For 2h-PBG, 21 studies were analyzed to compare the impact of different dietary interventions. **Figure 6.2** illustrates the direct comparisons between interventions and sample size distribution. According to **Supplementary Table 5.2**, the DASH diet significantly reduced 2h-PBG compared to the Low-GI diet (SMD = -1.41, 95% CI: -2.56 to -0.25), Low-GL diet (SMD = -1.84, 95% CI: -3.52 to -0.16), Low-carb diet (SMD = -2.21, 95% CIs: -3.89 to -0.53), and Standard diet (SMD = -2.21, 95% CIs: -3.32 to -1.10). Additionally, the Low-GI diet also significantly reduced 2h-PBG compared to the Standard diet (SMD = -0.81, 95% CIs: -1.12 to -0.49). SUCRA rankings (**Figure 7.2**) showed that the DASH diet (99.2%), Low-GI diet (65.9%), and Low-GL diet (41.1%) were the top interventions, with the Standard diet scoring the lowest at 19.5%. A forest plot summarizing these results is presented in **Figure 2**.

#### 3.2.3 HOMA-IR

Ten studies were included in the network meta-analysis of HOMA-IR to assess the effects of various dietary interventions on insulin resistance. **Figure 6.3** depicts the direct comparisons and sample size distribution. As indicated in **Supplementary Table 5.3**, the DASH diet significantly reduced HOMA-IR compared to the Low-GI diet (MD = -1.23, 95% CI: -1.78 to -0.67), Low-carb DASH diet (MD = -1.37, 95% CI: -2.12 to -0.63), Low-carb diet (MD = -1.76, 95% CI: -2.50 to -1.02), and Standard diet (MD = -1.90, 95% CI: -2.44 to -1.36). Additionally, the Low-GI diet significantly reduced HOMA-IR compared to the Low-carb diet (MD = -0.53, 95% CI: -1.06 to -0.01) and Standard diet (MD = -0.67, 95% CI: -0.80 to -0.54). In SUCRA rankings (**Figure 7.3**), the DASH diet (100.0%), Low-GI diet (67.3%), and Low-carb DASH diet (55.5%) were the top treatments, while the Standard diet scored the lowest at 7.8%. The forest plot for HOMA-IR is presented in **Figure 3**.

#### 3.2.4 Cesarean section

Ten studies were included in the network meta-analysis for cesarean section rates. **Figure 6.4** shows the direct comparisons and sample size distribution. As presented in **Supplementary Tables 5.4**, the DASH diet significantly reduced the risk of cesarean section compared to the Low-carb diet (RR = 0.56, 95% CI: 0.33 to 0.96) and the Standard diet (RR = 0.54, 95% CI: 0.40 to 0.74). SUCRA rankings (**Figure 7.4**) indicated that the top interventions for reducing cesarean section rates were the Low-GI diet (82.1%), Low-GL diet (74.9%), and DASH diet (68.2%), with the Standard diet scoring the lowest at 20.8%. The forest plot for cesarean section rates is available in **Figure 4.1**.

#### 3.2.5 Macrosomia

Twelve studies were analyzed to evaluate the impact of different dietary interventions on macrosomia rates. **Figure 6.5** displays the direct comparisons and sample size distribution. As shown in **Supplementary Table 5.5**, both the DASH diet (RR = 0.12, 95% CI: 0.03 to 0.51) and Low-GI diet (RR = 0.23, 95% CI: 0.10 to 0.54) significantly reduced the occurrence of macrosomia compared to the Standard diet. SUCRA rankings (**Figure 7.5**) revealed that the DASH diet (82.7%), Low-GI diet (66.0%), and Low-carb DASH diet (65.0%) were the top interventions, with the Standard diet scoring the lowest at 10.4%. The forest plot for macrosomia outcomes is presented in **Figure 4.2**.

#### 3.2.6 Gestational hypertension

Eight studies were included in the network meta-analysis for gestational hypertension. **Figure 6.6** presents the direct comparisons and sample size distribution. As indicated in **Supplementary Table 5.6**, the Low-GI diet significantly reduced the risk of gestational hypertension compared to the Standard diet (RR = 0.26, 95% CI: 0.11 to 0.65). SUCRA rankings (**Figure 7.6**) showed that the Low-GL diet (71.8%) and Low-GI diet (71.1%) were the top interventions for reducing gestational hypertension, with the Standard diet scoring the lowest at 7.1%. The corresponding forest plot is included in **Figure 4.3**.

#### 3.2.7 Preterm birth

Six studies were analyzed to assess the effects of dietary interventions on preterm birth rates. **Figure 6.7** illustrates the direct comparisons and sample size distribution. As shown in **Supplementary Table 5.7**, the Low-GI diet significantly reduced the risk of preterm birth compared to the Standard diet (RR = 0.48, 95% CI: 0.30 to 0.75). In terms of SUCRA rankings (**Figure 7.7**), the top interventions for reducing preterm birth were the Low-GI diet (85.8%) and Low-GL diet (50.4%), with the Standard diet having the lowest score at 13.7%. The forest plot summarizing these findings is presented in **Figure 4.4**.

### 3.3 Publication bias

Potential publication bias was evaluated using funnel plots (**Supplementary File 4**). The scatter plots displayed varying degrees of symmetry around the vertical axis, suggesting the possibility of publication bias. Specifically, **Supplementary Figures 4.3, 4.5, and 4.6** showed a fairly even distribution of points, indicating a lower likelihood of bias, whereas **Supplementary Figures 4.1, 4.2, 4.4**, and **4.7** demonstrated some degree of asymmetry, suggesting potential bias in these comparisons.

The Egger’s test for 2h-PBG yielded a P-value of 0.018 (P < 0.05), indicating a statistically significant result that warrants cautious interpretation. For the remaining outcomes, Egger’s test results were all above 0.05, suggesting no substantial evidence of publication bias across the overall analysis of the included studies.

### 3.4 Risk of bias

The risk of bias was assessed across all 28 studies. Thirteen studies were rated as having an overall low risk of bias, 11 studies were categorized as having some concerns, and 2 studies were deemed to have a high overall risk of bias. Specifically, in the domain of the randomization process, 20 studies were classified as low risk, while 8 studies had some concerns. Regarding deviations from intended interventions, 25 studies were rated as low risk, and 3 studies had some concerns. In the domain of missing outcome data, 22 studies were rated as low risk, 4 had some concerns, and 2 were classified as high risk. All 28 studies were rated as low risk in the measurement of outcomes, while 26 studies were classified as low risk for selective reporting, with 2 studies having some concerns. Further details are provided in **Supplementary File 3**.

### 3.5 GRADE assessment

The certainty of evidence was assessed using the GRADE framework across five domains. FBG was rated low due to some concerns in randomization and serious inconsistency (I^2^ = 96%, P < 0.01). No downgrades were made for indirectness, imprecision, or publication bias. 2h-PBG was rated very low due to concerns in randomization and missing outcome data, serious inconsistency (I^2^ = 87%, P < 0.01), and potential publication bias (Egger’s test, P < 0.05). Indirectness and imprecision remained unchanged. HOMA-IR was rated moderate, downgraded only for serious inconsistency (I^2^ = 94%, P < 0.01). Other domains remained intact. Cesarean Section was rated low due to randomization concerns and serious inconsistency (I^2^ = 82%, P < 0.01). No further downgrades. Macrosomia and Gestational Hypertension were rated moderate, each downgraded for randomization concerns, with no further downgrades. Preterm Birth was rated high, as no downgrades were applied, indicating strong evidence. For a detailed breakdown of the GRADE assessment, see **Supplementary File 3** (**Supplementary Figures 3.1–3.7**).

## 4 Discussion

This network meta-analysis of 28 RCTs comprehensively evaluated the effects of various dietary interventions on glycemic control and adverse pregnancy outcomes in patients with GDM. The findings revealed three key insights. First, in terms of glycemic control, the DASH diet was the most effective intervention, ranking highest for improving FBG, 2h-PBG, and HOMA-IR, compared to other dietary patterns. Second, regarding adverse pregnancy outcomes, the DASH and Low-GI diets significantly reduced complications such as cesarean section, macrosomia, and gestational hypertension, which are closely associated with poor glucose control and maternal metabolic dysregulation. Third, although the low-carbohydrate diet showed some efficacy in reducing FBG, it was less effective in improving 2h-PBG, insulin resistance, and reducing adverse pregnancy outcomes, potentially due to the lack of essential micronutrients and fiber. This suggests that diets with broader nutrient profiles, such as DASH and Low-GI, may offer additional benefits beyond glycemic control, likely due to their positive effects on insulin sensitivity, lipid metabolism, and vascular function.

In this study, the DASH diet was identified as the most effective intervention for glycemic control across all three key indicators: FBG, 2h-PBG, and HOMA-IR. This finding aligns with previous research, which has consistently demonstrated the benefits of the DASH diet in managing glycemic levels in both GDM and non-GDM populations. For instance, a study by Akhlaghi et al. found that GDM patients on the DASH diet experienced significant reductions in FBG and insulin resistance compared to those on standard diets (52). Similarly, Mahdavi et al. reported improvements in postprandial glucose levels and overall metabolic control in GDM patients following the DASH diet (53), emphasizing its role in controlling blood sugar levels without excessive dietary restrictions that may impact maternal nutrition. The mechanisms underlying the DASH diet’s superiority in glycemic control are likely due to several interrelated factors. Firstly, the diet’s high content of fiber-rich foods, such as whole grains, legumes, fruits, and vegetables, not only slows gastric emptying and carbohydrate absorption but also modulates gut microbiota, which plays a role in glucose homeostasis (54). This not only helps to maintain stable glucose levels but also reduces postprandial hyperglycemia, a critical issue in GDM management. Secondly, the DASH diet is rich in micronutrients, particularly magnesium and potassium, which have been shown to enhance insulin sensitivity (55, 56). Magnesium, in particular, plays a pivotal role in glucose transport, insulin receptor activity, and the prevention of oxidative stress-related insulin resistance (57). Moreover, the DASH diet’s emphasis on reducing saturated fat intake and increasing consumption of unsaturated fats contributes to improved lipid metabolism, vascular health, and reduced chronic inflammation, all of which are closely linked to insulin resistance (58). Research has shown that high-fat diets exacerbate insulin resistance, whereas the DASH diet’s balanced macronutrient and micronutrient composition mitigates these effects (52). Finally, the inclusion of lean proteins and low-fat dairy products, which are staples of the DASH diet, may further promote insulin sensitivity by modulating the incretin response and pancreatic β-cell function (14).

The reduction of adverse pregnancy outcomes is a critical focus in the management of GDM, as these complications can have long-term consequences for both the mother and the infant. In this study, the DASH and Low-GI diets were shown to significantly reduce the incidence of cesarean sections, macrosomia, and gestational hypertension, compared to other dietary interventions. These findings underscore the importance of dietary modifications not only for optimizing maternal glucose control but also for minimizing the risk of obstetric complications through metabolic and cardiovascular regulation. Previous studies have also highlighted the beneficial effects of these dietary patterns. For instance, research has demonstrated that the DASH diet, due to its high content of fiber, antioxidants, and lean proteins, helps manage weight gain during pregnancy, which is a key factor in preventing macrosomia and cesarean delivery (54). Similarly, Low-GI diets have been shown to stabilize blood glucose levels and reduce insulin fluctuations, which are closely linked to hypertensive disorders and fetal overgrowth during pregnancy. A study by Louie et al. found that Low-GI diets significantly reduced the risk of gestational hypertension and the need for cesarean sections in GDM patients (59). The mechanisms behind the protective effects of the DASH and Low-GI diets on adverse pregnancy outcomes are likely multifactorial. Both diets contribute to maternal vascular health, reducing oxidative stress and endothelial dysfunction, which are common in GDM and linked to pregnancy complications (30, 60). Additionally, the DASH diet’s emphasis on reducing sodium intake and increasing potassium-rich foods may contribute to improved blood pressure control, thereby lowering the risk of gestational hypertension (61). In the case of Low-GI diets, their role in reducing excessive fetal growth can be attributed to stable insulin secretion patterns, as high postprandial insulin levels have been associated with increased fetal adiposity and birth weight (62).

An interesting finding in this study was that, while the low-carbohydrate diet demonstrated some efficacy in reducing FBG, it was significantly less effective in improving 2h-PBG, insulin resistance, and reducing adverse pregnancy outcomes. This aligns with previous studies that have shown similar trends. For instance, a study by Rein et al. reported that although low-carbohydrate diets can result in significant reductions in FBG, they often fail to provide consistent improvements in postprandial glucose control and insulin sensitivity due to the rapid hepatic glucose output following protein and fat intake (63). Additionally, a meta-analysis by Snorgaard et al. found that low-carbohydrate diets were not as effective as low-glycemic index or Mediterranean-style diets in reducing pregnancy-related complications such as macrosomia and cesarean section rates, likely due to insufficient fiber intake and limited micronutrient diversity (64). The underlying mechanisms that explain the limited impact of low-carbohydrate diets on 2h-PBG and insulin resistance may be related to the quality and type of carbohydrates consumed rather than absolute carbohydrate restriction. While reducing carbohydrate intake lowers FBG, it does not necessarily improve postprandial glucose control if the remaining carbohydrates are of high glycemic index, leading to glucose fluctuations (12). Furthermore, the relatively high intake of proteins and fats in low-carbohydrate diets can lead to a compensatory increase in hepatic gluconeogenesis and free fatty acid oxidation, potentially exacerbating insulin resistance over time, as suggested by research showing that diets high in saturated fats negatively affect insulin sensitivity (65). This dietary pattern may also lack the beneficial bioactive compounds, prebiotics, and polyphenols found in more balanced diets like DASH and Low-GI, which are essential for reducing systemic inflammation and improving pancreatic β-cell function (66).

This study has several strengths. First, it employed a robust network meta-analysis approach, which allows for the comparison of multiple dietary interventions simultaneously, providing a hierarchical ranking of efficacy based on direct and indirect evidence. This method also enabled indirect comparisons between interventions that had not been directly compared in previous trials, offering valuable insights into the optimal dietary strategies for GDM management. Second, the inclusion of high-quality RCTs enhanced the reliability of the findings, as RCTs are considered the gold standard for evaluating intervention effectiveness. However, there are also several limitations to consider. One important limitation is the heterogeneity in routine dietary interventions within the standard care groups, which may have influenced the observed effect sizes. Differences in the composition and quality of standard dietary advice provided to control groups could not be fully standardized, potentially affecting comparative efficacy estimates. While a random-effects model was used to account for this, baseline dietary intake variability and adherence rates across studies remain a confounding factor. Additionally, sensitivity analyses were conducted to assess the impact of this heterogeneity on our findings, confirming the robustness of our primary results. Second, the certainty of evidence for several outcomes was low to moderate, as evaluated using the GRADE framework. While the evidence for preterm birth was rated as high certainty, the certainty of evidence for HOMA-IR, macrosomia, and gestational hypertension was moderate, whereas FBG and cesarean section were rated as low certainty, and 2h-PBG as very low certainty. Given that only 13 out of 28 studies had an overall low risk of bias, factors such as inconsistent randomization protocols, missing outcome data, and publication bias may have influenced the certainty of evidence. Third, variability in dietary protocols across studies, including differences in meal composition, macronutrient distribution, and adherence monitoring, may have contributed to heterogeneity. While a random-effects model was applied to address this, discrepancies in intervention intensity, duration, and participant adherence were not uniformly reported, which may have affected the robustness of effect estimates. Fourth, small sample sizes in several included studies may have limited the statistical power of certain comparisons, particularly for rare adverse pregnancy outcomes such as preterm birth and severe neonatal complications. Future studies with larger sample sizes and longer follow-up durations are needed to validate the observed effects and assess long-term maternal and fetal health outcomes. Fifth, publication bias remains a concern, as studies with positive results are more likely to be published. Although funnel plot analysis and Egger’s test were employed to assess this, some outcomes, such as 2h-PBG, showed potential signs of bias. This highlights the need for pre-registering study protocols and ensuring the publication of null findings to improve the reliability of future evidence.

## 5 Conclusion

This network meta-analysis suggests that DASH and Low-GI diets may offer promising benefits for glycemic control and reducing adverse pregnancy outcomes in patients with gestational diabetes mellitus. The DASH diet showed favorable trends for improving fasting and postprandial glucose levels, while both diets appeared to mitigate risks of macrosomia, cesarean delivery, and gestational hypertension. However, due to variability in study quality and design, these findings warrant cautious interpretation. Further large-scale, methodologically rigorous trials are needed to confirm optimal dietary strategies and refine clinical guidance for gestational diabetes management.

## Data Availability

The original contributions presented in the study are included in the article/**Supplementary Material**. Further inquiries can be directed to the corresponding author.
