# Supplementary material for: Comparative efficacy of dietary interventions for glycemic control and pregnancy outcomes in gestational diabetes: a network meta-analysis of randomized controlled trials
Source: Front Endocrinol (Lausanne). 2025 May 19;16:1512493. doi: 10.3389/fendo.2025.1512493 (PMC12127149; doi:10.3389/fendo.2025.1512493)
Supplement: Supplementary file 1 [file DataSheet1.docx]

**Supplementary**

Table of Contents:

[Supplementary 1: Search Strategy 2](#_Toc159076731)

[Supplementary 2: Characteristics of studies and subjects included in the review 4](#_Toc159076743)

[Supplementary 3: Risk of Bias 1](#_Toc159076747)5

[Supplementary 4: Publication bias](#_Toc159076752) 24

[Supplementary 5: League table of outcomes](#_Toc159076747) 31

# Supplementary 1: Search Strategy

***Search Strategy:***

| #1 | Search: (Diabetes, Gestational [MeSH Terms]) |
| --- | --- |
| #2 | Search: (Diabetes Mellitus, Gestational [Title/Abstract]) OR (Gestational Diabetes Mellitus [Title/Abstract]) OR (Gestational Diabetes [Title/Abstract]) |
| #3 | Search: ((((Diabetes, Gestational [MeSH Terms]) OR (Diabetes Mellitus, Gestational [Title/Abstract]) OR (Gestational Diabetes Mellitus [Title/Abstract]) OR (Gestational Diabetes [Title/Abstract]) |
| #4 | Search: (Fasting Blood Glucose [MeSH Terms]) |
| #5 | Search: ((FBG [Title/Abstract]) OR ("Fasting Glucose" [Title/Abstract]) |
| #6 | Search: (((Fasting Blood Glucose [MeSH Terms]) OR (FBG [Title/Abstract]) OR ("Fasting Glucose" [Title/Abstract]) |
| #7 | Search: ("2-Hour Postprandial Blood Glucose" [Title/Abstract]) OR ("Postprandial Glucose" [Title/Abstract]) OR (2h-PBG [Title/Abstract]) |
| #8 | Search: (Insulin Resistance [MeSH Terms]) |
| #9 | Search: ("HOMA-IR" [Title/Abstract]) OR ("Insulin Resistance Index" [Title/Abstract]) |
| #10 | Search: (Insulin Resistance [MeSH Terms]) OR ("HOMA-IR" [Title/Abstract]) OR ("Insulin Resistance Index" [Title/Abstract]) |
| #11 | Search: (Pregnancy Outcome [MeSH Terms]) |
| #12 | Search: ((((("Adverse Pregnancy Outcomes" [Title/Abstract]) OR (preeclampsia [Title/Abstract]) OR ("preterm birth" [Title/Abstract]) OR (macrosomia [Title/Abstract]) OR ("neonatal hypoglycemia" [Title/Abstract]) |
| #13 | Search: ((((((Pregnancy Outcome [MeSH Terms]) OR ("Adverse Pregnancy Outcomes" [Title/Abstract]) OR (preeclampsia [Title/Abstract]) OR ("preterm birth" [Title/Abstract]) OR (macrosomia [Title/Abstract]) OR ("neonatal hypoglycemia" [Title/Abstract]) |
| #14 | Search: ((((((((((((Fasting Blood Glucose [MeSH Terms]) OR (FBG [Title/Abstract]) OR ("Fasting Glucose" [Title/Abstract]) OR ("2-Hour Postprandial Blood Glucose" [Title/Abstract]) OR ("Postprandial Glucose" [Title/Abstract]) OR (2h-PBG [Title/Abstract]) OR (Insulin Resistance [MeSH Terms]) OR ("HOMA-IR" [Title/Abstract]) OR ("Insulin Resistance Index" [Title/Abstract]) OR ((((((Pregnancy Outcome [MeSH Terms]) OR ("Adverse Pregnancy Outcomes" [Title/Abstract]) OR (preeclampsia [Title/Abstract]) OR ("preterm birth" [Title/Abstract]) OR (macrosomia [Title/Abstract]) OR ("neonatal hypoglycemia" [Title/Abstract]) |
| #15 | Search: ((((Dietary Intervention [Title/Abstract]) OR ("nutritional therapy" [Title/Abstract]) OR ("Low Glycemic Index Diet" [Title/Abstract]) OR ("Low Glycemic Load Diet" [Title/Abstract]) |
| #15 | Search: (((((((randomized controlled trial[pt] OR controlled clinical trial[pt] OR randomized[tiab] OR  placebo[tiab] OR drug therapy[sh] OR randomly[tiab] OR trial[tiab] OR groups[tiab]) NOT (animals[mh] NOT humans[mh]) |
| #16 | Search: ((((Diabetes, Gestational [MeSH Terms]) OR (Diabetes Mellitus, Gestational [Title/Abstract]) OR (Gestational Diabetes Mellitus [Title/Abstract]) OR (Gestational Diabetes [Title/Abstract]) AND ((((((((((((Fasting Blood Glucose [MeSH Terms]) OR (FBG [Title/Abstract]) OR ("Fasting Glucose" [Title/Abstract]) OR ("2-Hour Postprandial Blood Glucose" [Title/Abstract]) OR ("Postprandial Glucose" [Title/Abstract]) OR (2h-PBG [Title/Abstract]) OR (Insulin Resistance [MeSH Terms]) OR ("HOMA-IR" [Title/Abstract]) OR ("Insulin Resistance Index" [Title/Abstract]) OR ((((((Pregnancy Outcome [MeSH Terms]) OR ("Adverse Pregnancy Outcomes" [Title/Abstract]) OR (preeclampsia [Title/Abstract]) OR ("preterm birth" [Title/Abstract]) OR (macrosomia [Title/Abstract]) OR ("neonatal hypoglycemia" [Title/Abstract]) AND ((((Dietary Intervention [Title/Abstract]) OR ("nutritional therapy" [Title/Abstract]) OR ("Low Glycemic Index Diet" [Title/Abstract]) OR ("Low Glycemic Load Diet" [Title/Abstract]) AND (((((((randomized controlled trial[pt] OR controlled clinical trial[pt] OR randomized[tiab] OR  placebo[tiab] OR drug therapy[sh] OR randomly[tiab] OR trial[tiab] OR groups[tiab]) NOT (animals[mh] NOT humans[mh]) |

# Supplementary 2: Characteristics of studies and subjects included in the review

| **Study** | **Country/Region** | **Subjects  (intervention/ control)** | **Mean age  (intervention/ control)** | **Type of disease** | **Baseline fasting blood glucose level** | **Pre-pregnancy BMI** | **Intervention detail** | | **Outcomes** |
| --- | --- | --- | --- | --- | --- | --- | --- | --- | --- |
|  |  |  |  |  |  |  | **Intervention group** | **Control group** |  |
| Cypryk et al. (2007) ^[24]^ | Poland | 30 (15/15) | 28.7±3.7 | Gestational Diabetes Mellitus (GDM) | 82±10 mg/dl vs. 77±8 mg/dl | NA | Low GI diet: Carbohydrates 45% of total energy, protein 25%, fat 30%. | Standard diet: Carbohydrates 65%, protein 25%, fat 10% of total energy intake. | Fasting blood glucose level, 2-hour postprandial blood glucose level |
| Hu et al. (2014) ^[31]^ | China | 140 (66/74) | 30.3±4.9 vs. 29.7±3.7 | Gestational Diabetes Mellitus (GDM) | 5.09±0.78 mmol/L vs. 4.99±0.56 mmol/L | 21.2±2.5 kg/m² vs. 20.9±3.4 kg/m² | Low GI diet: Low-GI staples replace regular rice, with GI values <55. | Standard diet: A typical diabetes control diet with white rice as the staple food. | Fasting blood glucose level, 2-hour postprandial blood glucose level |
| Louie et al. (2011) ^[26]^ | Australia | 99 (50/49) | 34.0±4.1 vs. 32.4±4.5 | Gestational Diabetes Mellitus (GDM) | 4.7±0.1 mmol/L vs. 4.6±0.1 mmol/L | 23.9±4.4 kg/m² vs. 24.1±5.7 kg/m² | Low GI diet: Target GI value: 50, carbohydrate intake: 40-45%. | Standard diet: Target GI value: 60, carbohydrate intake: 40-45%. | Fasting blood glucose level, |
| Asemi et al. (2013) ^[27]^ | Iran | 32 (16/16) | 27.7±5.4 vs. 29.7±5.6 | Gestational Diabetes Mellitus (GDM) | 92.43±2.77 mg/dL vs. 88.75±2.87 mg/dL | 30.2±4.6 kg/m² vs. 29.7±3.3 kg/m² | DASH diet: High in fruits, vegetables, whole grains, and low-fat dairy; low in saturated and total fats, cholesterol, refined grains, and sweets; sodium ≤2400 mg/day. | Standard diet: Carbohydrates 40-55% of energy, protein 10-20%, fat 25-30%. | Fasting blood glucose level, 2-hour postprandial blood glucose level |
| Grant et al. (2011) ^[25]^ | Canada | 47 (24/23) | 34.0±1.1 vs. 34.0±1.1 | Gestational Diabetes Mellitus (GDM) | 4.5±0.2 mmol/L vs. 5.0±0.2 mmol/L | 27±1 kg/m² vs. 26±1 kg/m² | Low GI diet: Consume foods with a GI <50, limit high-GI foods. | Standard diet: Consume foods with a higher GI (approximately 58). | Fasting blood glucose level, 2-hour postprandial blood glucose level |
| Jie et al. (2015) ^[32]^ | China | 33 (17/16) | 30.7±5.6 vs. 28.3±5.1 | Gestational Diabetes Mellitus (GDM) | 97.9±14.2 mg/dL vs. 98.1±12.3 mg/dL | 30.2±4.1 kg/m² vs. 30.9±3.6 kg/m² | DASH diet: High in fruits, vegetables, whole grains, and low-fat dairy; low in saturated and total fats, cholesterol, refined grains, and sweets; sodium ≤2400 mg/day. | Standard diet: Carbohydrates 40-55% of energy, protein 10-20%, fat 25-30%. | Fasting blood glucose level, Insulin resistance index, Cesarean section rate, Incidence of macrosomia |
| Allehdan et al. (2022) ^[43]^ | Jordan | 70 (23/23/24) | 34.0±4.3 vs. 33.1±5.0 vs. 33.6±4.9 | Gestational Diabetes Mellitus (GDM) | 91.0±11.0 mg/dL vs. 95.0±14.1 mg/dL vs. 94.1±15.7 mg/dL | 29.3±6.0 kg/m² vs. 29.3±7.2 kg/m² vs. 29.7±6.3 kg/m² | (a) Carbohydrate diet: Plan meals and three snacks using carbohydrate counting. (b) Carbohydrate-DASH diet: Combine carbohydrate counting with DASH diet principles, emphasizing fruits, vegetables, whole grains, and low-fat dairy, while reducing saturated fats, cholesterol, and sodium intake (<2400 mg/day). | Standard diet: A standard diet with 45-55% carbohydrates, 15-20% protein, and 25-30% fat. | Fasting blood glucose level, Insulin resistance index, Cesarean section rate, Incidence of macrosomia |
| Dodesini et al. (2021) ^[41]^ | Italy | 32 (16/16) | 35±5 | Gestational Diabetes Mellitus (GDM) | 82±5 mg/dL vs. 88±4 mg/dL | 26±6 kg/m² | Low GI diet: GI ≤50, fiber intake ≥35 g/day. | Standard diet: GI >50, fiber intake <35 g/day. | Fasting blood glucose level, |
| Mijatovic et al. (2020) ^[40]^ | Australia | 46 (24/22) | 32.5±0.9 vs. 34.2±0.9 | Gestational Diabetes Mellitus (GDM) | 4.8±0.1 mmol/L vs. 4.7±0.1 mmol/L | 25.8±1.0 kg/m² vs. 27.8±1.5 kg/m² | Low-carb diet: Target 135g carbohydrates/day, no restriction on total energy intake. | Standard diet: Carbohydrate intake target: 180-200g/day. | Fasting blood glucose level, Cesarean section rate, Incidence of macrosomia |
| Lv et al. (2019) ^[37]^ | China | 134 (67/67) | NA | Gestational Diabetes Mellitus (GDM) | 6.71±0.61 mmol/L vs. 6.68±0.57 mmol/L | NA | Low GL diet: Adjust intake based on GL, limit high-GL foods (>20), favor low-GL foods (<10). | Standard diet: Nutritional intervention with traditional food exchange method, without considering glycemic load. | Fasting blood glucose level, 2-hour postprandial blood glucose level, Cesarean section rate, Incidence of macrosomia, Preeclampsia |
| Asemi et al. (2013) ^[28]^ | Iran | 34 (17/17) | 30.7±6.7 vs. 29.4±6.2 | Gestational Diabetes Mellitus (GDM) | 5.25±0.94 mmol/L vs. 5.26±0.74 mmol/L | 29.0±3.2 kg/m² vs. 31.4±5.7 kg/m² | DASH diet: High in fruits, vegetables, whole grains, and low-fat dairy; low in saturated and total fats, cholesterol, refined grains, and sweets; sodium ≤2400 mg/day. | Standard diet: Carbohydrates 40-55% of energy, protein 10-20%, fat 25-30%. | Fasting blood glucose level, 2-hour postprandial blood glucose level, Cesarean section rate |
| Ma et al. (2014) ^[33]^ | China | 83 (41/42) | 30.1±3.8 vs. 30.0±3.5 | Gestational Diabetes Mellitus (GDM) | 4.96±0.81 mmol/L vs. 4.80±0.55 mmol/L | 21.90±3.14 kg/m² vs. 21.15±2.75 kg/m² | Low GI diet | Standard diet | Fasting blood glucose level, 2-hour postprandial blood glucose level, Cesarean section rate, Incidence of macrosomia, Preterm birth |
| Moreno-Castilla et al. (2013) ^[29]^ | Spanish | 150 (75/75) | 33.5±3.7 vs. 32.1±4.4 | Gestational Diabetes Mellitus (GDM) | 4.9±0.7 mmol/L vs. 5.0±0.5 mmol/L | 25.4±5.7 kg/m² vs. 26.6±5.5 kg/m² | Low-carb diet: 40% of total calories from carbohydrates, 20% from protein, 40% from fat. | Standard diet: 55% of total calories from carbohydrates, 20% from protein, 25% from fat. | 2-hour postprandial blood glucose level |
| Asemi et al. (2014) ^[30]^ | Iran | 52 (26/26) | 31.9±6.1 vs. 30.7±6.3 | Gestational Diabetes Mellitus (GDM) | 96.4±15 mg/dL vs. 95.3±12.6 mg/dL | 29.2±3.5 kg/m² vs. 31.0±4.9 kg/m² | DASH diet: High in fruits, vegetables, whole grains, and low-fat dairy; low in saturated and total fats, cholesterol, refined grains, and sweets; sodium ≤2400 mg/day. | Standard diet: Carbohydrates 40-55% of energy, protein 10-20%, fat 25-30%. | Cesarean section rate, Incidence of macrosomia |
| Hernandez et al. (2016) ^[34]^ | USA | 12 (6/6) | 28±2 vs. 30±1 | Gestational Diabetes Mellitus (GDM) | 82±3.5 mg/dL vs. 79.3±2.4 mg/dL | 33.4±1.4 kg/m² vs. 34.3±1.6 kg/m² | Low-carb diet: 40% carbs, 45% fat, 15% protein. | Standard diet: High complex-carb, low-fat (60% carbs, 25% fat, 15% protein). | Fasting blood glucose level, Insulin resistance index |
| Xiaoqiong et al. (2024) ^[48]^ | China | 566 (283/283) | 29.17±2.18 vs. 28.81±2.47 | Gestational Diabetes Mellitus (GDM) | 7.52±1.13 mmol/L vs. 7.39±1.36 mmol/L | NA | Low-GI diet: Low glycemic index dietary intervention, GI <55. | Standard diet: Carbohydrates ~50%, fat ~30%, protein ~20%. | Fasting blood glucose level, 2-hour postprandial blood glucose level, Insulin resistance index, Preterm birth |
| Linna et al. (2020) ^[39]^ | China | 128 (64/64) | 29.32±4.62 vs. 30.14±3.52 | Gestational Diabetes Mellitus (GDM) | 5.99±0.24 mmol/L vs. 6.02±0.35 mmol/L | NA | Low-GI diet: Low glycemic index dietary intervention, GI <55. | Standard diet: 50-60% carbohydrates, 20% protein, 20-30% fat. | Fasting blood glucose level, 2-hour postprandial blood glucose level, Insulin resistance index, Preeclampsia, Preterm birth |
| Hongru et al. (2020) ^[38]^ | China | 80 （40/40） | 29.18±4.70 vs. 29.32±4.62 | Gestational Diabetes Mellitus (GDM) | 9.08±1.52 mmol/L vs.9.12±1.54 mmol/L | NA | Low-GI diet: Low glycemic index dietary intervention, GI <55. | Standard diet: 50-60% carbohydrates, 20% protein, 20-30% fat. | Fasting blood glucose level, 2-hour postprandial blood glucose level, Insulin resistance index, Incidence of macrosomia |
| Zhuqing et al. (2024) ^[51]^ | China | 86 (43/43) | 30.19±2.20 vs. 30.20±2.22 | Gestational Diabetes Mellitus (GDM) | 8.29±0.71 mmol/L vs. 8.31±0.72 mmol/L | NA | Low-GI diet: Low glycemic index dietary intervention, GI <55. | Standard diet: 50-60% carbohydrates, 20% protein, 20-30% fat. | Fasting blood glucose level, 2-hour postprandial blood glucose level, Insulin resistance index, Cesarean section rate, Incidence of macrosomia |
| Fengying et al. (2019) ^[36]^ | China | 136 (68/68) | 26.39±3.27 vs. 26.09±3.10 | Gestational Dysglycemia | 4.89±1.15 mmol/L vs. 5.78±1.23 mmol/L | 23.57±2.35 kg/m² vs. 23.33±2.08 kg/m² | Low-GI diet: Low glycemic index dietary intervention, GI <55. | Standard diet: 50-60% carbohydrates, 20% protein, 20-30% fat. | Fasting blood glucose level, 2-hour postprandial blood glucose level, Incidence of macrosomia |
| Liyu et al. (2022) ^[45]^ | China | 80 (40/40) | 30.59±1.31 vs. 30.52±1.28 | Gestational Diabetes Mellitus (GDM) | 7.61±1.51 mmol/L vs. 7.64±1.45 mmol/L | NA | Low-GI diet: Low glycemic index dietary intervention, GI <55. | Standard diet: 50-60% carbohydrates, 20% protein, 20-30% fat. | Fasting blood glucose level, 2-hour postprandial blood glucose level, Cesarean section rate, Preterm birth |
| Ke et al. (2022) ^[44]^ | China | 92 (46/46) | 30.26±3.44 vs. 30.22±3.41 | Gestational Diabetes Mellitus (GDM) | 7.65±1.59 mmol/L vs. 7.62±1.62 mmol/L | NA | Low-GI diet | Standard diet | Fasting blood glucose level, 2-hour postprandial blood glucose level, Preeclampsia |
| Ying et al. (2023) ^[49]^ | China | 90 (45/45) | 28.69±2.77 vs. 29.60±2.06 | Gestational Diabetes Mellitus (GDM) | 6.10±0.51 mmol/L vs. 6.14±0.60 mmol/L | 22.58±0.96 kg/m² vs. 23.23±0.75 kg/m² | Low-GI diet | Standard diet | Fasting blood glucose level, 2-hour postprandial blood glucose level, Incidence of macrosomia, Preeclampsia |
| Bo et al. (2017) ^[35]^ | China | 60 (30/30) | 32.01±1.05 vs. 31.57±1.22 | Gestational Diabetes Mellitus (GDM) | 7.3±0.8 mmol/L vs. 7.1±0.6 mmol/L | NA | Low-GI diet | Standard diet | Fasting blood glucose level, 2-hour postprandial blood glucose level, Preeclampsia |
| Lixia et al. (2022) ^[46]^ | China | 78 (39/39) | NA | Gestational Diabetes Mellitus (GDM) | 6.13±0.51 mmol/L vs. 6.06±0.48 mmol/L | NA | Low-GI diet: Controlled carbohydrate intake, selection of low-GI foods. | Standard diet | Fasting blood glucose level, 2-hour postprandial blood glucose level, Insulin resistance index |
| Yuman et al. (2023) ^[50]^ | China | 104 (52/52) | 29.90±2.01 vs. 30.02±1.72 | Gestational Diabetes Mellitus (GDM) | 6.10±0.54 mmol/L vs. 6.14±0.60 mmol/L | 23.02±0.74 kg/m² vs. 22.98±0.82 kg/m² | Low-GI diet: Low glycemic index dietary intervention, GI <55. | Standard diet: 50-60% carbohydrates, 20% protein, 20-30% fat. | Fasting blood glucose level, 2-hour postprandial blood glucose level, Insulin resistance index, Incidence of macrosomia, Preeclampsia, Preterm birth |
| Wen et al. (2022) ^[47]^ | China | 110 (55/55) | 28.88±2.85 vs. 28.11±3.21 | Gestational Diabetes Mellitus (GDM) | 7.17±0.85 mmol/L vs. 7.13±0.82 mmol/L | NA | Low-GI diet | Standard diet | Fasting blood glucose level, 2-hour postprandial blood glucose level, Insulin resistance index, Preeclampsia, Preterm birth |
| Yan et al. (2021) ^[42]^ | China | 62 (31/31) | 31.72±2.36 vs. 31.69±2.13 | Gestational Diabetes Mellitus (GDM) | 7.30±0.42 mmol/L vs. 7.41±0.40 mmol/L | NA | Low-GI diet: 175g carbohydrates/day, ≤20% fat intake, protein from white meat and seafood. | Standard diet: Choose moderate-GI meals with GI 55-75. | Fasting blood glucose level, 2-hour postprandial blood glucose level, Cesarean section rate, Incidence of macrosomia, Preeclampsia |

Note: BMI, Body Mass Index, NA, Not Applicable.

**List of included studies:**

1. Cypryk K, Kamińska P, Kosiński M, Pertyńska-Marczewska M, Lewiński A. A comparison of the effectiveness, tolerability and safety of high and low carbohydrate diets in women with gestational diabetes. Endokrynol Pol. 2007 Jul-Aug;58(4):314-9. PMID: 18058723.
2. Hu ZG, Tan RS, Jin D, Li W, Zhou XY. A low glycemic index staple diet reduces postprandial glucose values in Asian women with gestational diabetes mellitus. J Investig Med. 2014 Dec;62(8):975-9. doi: 10.1097/JIM.0000000000000108. PMID: 25203150.
3. Louie JC, Markovic TP, Perera N, Foote D, Petocz P, Ross GP, Brand-Miller JC. A randomized controlled trial investigating the effects of a low-glycemic index diet on pregnancy outcomes in gestational diabetes mellitus. Diabetes Care. 2011 Nov;34(11):2341-6. doi: 10.2337/dc11-0985. Epub 2011 Sep 6. PMID: 21900148; PMCID: PMC3198285.
4. Asemi Z, Samimi M, Tabassi Z, Sabihi SS, Esmaillzadeh A. A randomized controlled clinical trial investigating the effect of DASH diet on insulin resistance, inflammation, and oxidative stress in gestational diabetes. Nutrition. 2013 Apr;29(4):619-24. doi: 10.1016/j.nut.2012.11.020. PMID: 23466048.
5. Grant SM, Wolever TM, O'Connor DL, Nisenbaum R, Josse RG. Effect of a low glycaemic index diet on blood glucose in women with gestational hyperglycaemia. Diabetes Res Clin Pract. 2011 Jan;91(1):15-22. doi: 10.1016/j.diabres.2010.09.002. Epub 2010 Nov 20. PMID: 21094553.
6. Jie Y , Lin C , Benli Z ,et al.Effect of dietary approaches to stop hypertension diet plan on pregnancy outcome patients with gestational diabetes mellitus[J].Bangladesh Journal of Pharmacology, 2015, 10(4):732.DOI:10.3329/bjp.v10i4.23813.
7. Allehdan S, Basha A, Hyassat D, Nabhan M, Qasrawi H, Tayyem R. Effectiveness of carbohydrate counting and Dietary Approach to Stop Hypertension dietary intervention on managing Gestational Diabetes Mellitus among pregnant women who used metformin: A randomized controlled clinical trial. Clin Nutr. 2022 Feb;41(2):384-395. doi: 10.1016/j.clnu.2021.11.039. Epub 2021 Dec 3. PMID: 34999333.
8. Dodesini AR, Donadoni V, Ciriello E, Colombo O, Patanè L, Galliani S, Cortinovis F, Trevisan R. Effects of a low glycemic index high in fiber diet on blood glucose in women with gestational diabetes. J Matern Fetal Neonatal Med. 2021 Mar;34(6):1010-1011. doi: 10.1080/14767058.2019.1622674. Epub 2019 Jun 3. PMID: 31154907.
9. Mijatovic J, Louie JCY, Buso MEC, Atkinson FS, Ross GP, Markovic TP, Brand-Miller JC. Effects of a modestly lower carbohydrate diet in gestational diabetes: a randomized controlled trial. Am J Clin Nutr. 2020 Aug 1;112(2):284-292. doi: 10.1093/ajcn/nqaa137. PMID: 32537643.
10. Lv S, Yu S, Chi R, Wang D. Effects of nutritional nursing intervention based on glycemic load for patient with gestational diabetes mellitus. Ginekol Pol. 2019;90(1):46-49. doi: 10.5603/GP.2019.0007. PMID: 30756370.
11. Asemi Z, Tabassi Z, Samimi M, Fahiminejad T, Esmaillzadeh A. Favourable effects of the Dietary Approaches to Stop Hypertension diet on glucose tolerance and lipid profiles in gestational diabetes: a randomised clinical trial. Br J Nutr. 2013 Jun;109(11):2024-30. doi: 10.1017/S0007114512004242. Epub 2012 Nov 13. PMID: 23148885.
12. Ma WJ, Huang ZH, Huang BX, Qi BH, Zhang YJ, Xiao BX, Li YH, Chen L, Zhu HL. Intensive low-glycaemic-load dietary intervention for the management of glycaemia and serum lipids among women with gestational diabetes: a randomized control trial. Public Health Nutr. 2015 Jun;18(8):1506-13. doi: 10.1017/S1368980014001992. Epub 2014 Sep 15. PMID: 25222105; PMCID: PMC10271623.
13. Moreno-Castilla C, Hernandez M, Bergua M, Alvarez MC, Arce MA, Rodriguez K, Martinez-Alonso M, Iglesias M, Mateu M, Santos MD, Pacheco LR, Blasco Y, Martin E, Balsells N, Aranda N, Mauricio D. Low-carbohydrate diet for the treatment of gestational diabetes mellitus: a randomized controlled trial. Diabetes Care. 2013 Aug;36(8):2233-8. doi: 10.2337/dc12-2714. Epub 2013 Apr 5. PMID: 23564917; PMCID: PMC3714525.
14. Asemi Z, Samimi M, Tabassi Z, Esmaillzadeh A. The effect of DASH diet on pregnancy outcomes in gestational diabetes: a randomized controlled clinical trial. Eur J Clin Nutr. 2014 Apr;68(4):490-5. doi: 10.1038/ejcn.2013.296. Epub 2014 Jan 15. PMID: 24424076.
15. Hernandez TL, Van Pelt RE, Anderson MA, Reece MS, Reynolds RM, de la Houssaye BA, Heerwagen M, Donahoo WT, Daniels LJ, Chartier-Logan C, Janssen RC, Friedman JE, Barbour LA. Women With Gestational Diabetes Mellitus Randomized to a Higher-Complex Carbohydrate/Low-Fat Diet Manifest Lower Adipose Tissue Insulin Resistance, Inflammation, Glucose, and Free Fatty Acids: A Pilot Study. Diabetes Care. 2016 Jan;39(1):39-42. doi: 10.2337/dc15-0515. Epub 2015 Jul 29. PMID: 26223240; PMCID: PMC4686845.
16. Zhang X, Yang F, Xiao L. Effects of Low Glycemic Index Dietary Intervention on Glycemic Control and Pregnancy Outcomes in Patients with Gestational Diabetes Mellitus. Diabetes New World. 2022 Sep;(9):1-5. doi: 10.16658/j.cnki.1672-4062.2022.18.001.
17. Wu L, Jia R. Effect of low glycemic index diet on glucose and lipid metabolism and maternal and infant outcomes in patients with gestational diabetes. Hainan Med J. 2020 Feb;31(4):455-458. doi: 10.3969/j.issn.1003-6350.2020.04.013.
18. Zhang H, Guo Y. Application effect of low-glycemic index dietary in pregnant women with gestational diabetes mellitus. Clin Med. 2020 Apr;(4):78-80. doi: 10.19347/j.cnki.2096-1413.202011035.
19. Huang Z. Intervention Effect Analysis of Low Glycemic Index Diet on Nutritional Treatment of Pregnancy Diabetes. Med Pract. 2024;8:123-130. doi: 10.19335/j.cnki.2096-1219.2024.08.030.
20. Lu F, Huang C, Ao D. The Effect of Dietary Intervention with Hypoglycemic Index on Maternal and Fetal Outcomes of Abnormal Glucose Metabolism in Pregnancy. Chinese and Foreign Medical Research. 2019 Sep;17(26):172-174. doi: 10.14033/j.cnki.cfmr.2019.26.076.
21. Wei L. Application Value of Low Glycemic Index Diet in Nutritional Treatment of Gestational Diabetes Mellitus and Analysis of Its Effect on Blood Glucose Control. Diabetes New World. 2022 Nov;(11):181-184. doi: 10.16658/j.cnki.1672-4062.2022.22.181.
22. Ke K, Dong J. The Application Value of Low Glycemic Index Diet in Nutritional Treatment of Gestational Diabetes Mellitus and Its Effect on Blood Glucose Control. Contemporary Medicine. 2022 Feb;28(4):139-140. doi: 10.3969/j.issn.1009-4393.2022.04.050.
23. Li Y. Application value of low glycemic index diet in nutritional treatment of diabetes in pregnancy. Electron J Pract Gynecol Endocrinol. 2023;10(34):45-47. doi: 10.3969/j.issn.2095-8803.2023.34.014.
24. Wang B. Application Value of Low Glycemic Index Diet in Nutritional Treatment of Gestational Diabetes Mellitus. Diabetes New World. 2017 Apr;(4):37-38. doi: 10.16658/j.cnki.1672-4062.2017.08.037.
25. Yao L. Analysis of the Value of Low Glycemic Index Diet in the Nutritional Treatment of Gestational Diabetes Mellitus. Diabetes New World. 2022 Dec;(12):58-61. doi: 10.16658/j.cnki.1672-4062.2022.23.058.
26. Sun Y, Wang Y. Application value of low glycemic index diet in nutritional treatment of gestational diabetes mellitus. Electron J Pract Gynecol Endocrinol. 2023;10(15):37-39. doi: 10.3969/j.issn.2095-8803.2023.15.011.
27. Tu W. The application value of low-glycemic index diet in nutritional treatment of gestational diabetes mellitus. Chin J Mod Drug Appl. 2022 Mar;16(6):234-236. doi: 10.14164/j.cnki.cn11-5581/r.2022.06.090.
28. Mu Y. Analysis of the Effect of Diet with Low Glycemic Index in the Nutritional Treatment of Gestational Diabetes. Diabetes New World. 2021 Feb;(2):43-45. doi: 10.16658/j.cnki.1672-4062.2021.04.043.

# Supplementary 3: Risk of Bias

## Table 3.1 The risk of bias assessment for the individual included studies

| **Study** | **Randomization process** | **Blinding of participants and personnel** | **Deviations from intended interventions** | **Missing outcome data** | **Measurement of the outcome** | **Selection of the reported result** | **Overall Bias** |
| --- | --- | --- | --- | --- | --- | --- | --- |
| Cypryk et al. (2007) ^[24]^ | Low | Some concerns | Some concerns | Low | Low | Low | Some concerns |
| Hu et al. (2014) ^[31]^ | Some concerns | Some concerns | Low | Low | Low | Low | Some concerns |
| Louie et al. (2011) ^[26]^ | Some concerns | Some concerns | Low | Low | Low | Low | Some concerns |
| Asemi et al. (2013) ^[27]^ | Low | Some concerns | Low | High | Low | Low | High |
| Grant et al. (2011) ^[25]^ | Low | Some concerns | Low | Low | Low | Some concerns | Some concerns |
| Jie et al. (2015) ^[32]^ | Low | Low | Low | Low | Low | Low | Low |
| Allehdan et al. (2022) ^[43]^ | Low | Low | Low | Low | Low | Low | Low |
| Dodesini et al. (2021) ^[41]^ | Some concerns | Some concerns | Low | Low | Low | Low | Some concerns |
| Mijatovic et al. (2020) ^[40]^ | Low | Some concerns | Low | Some concerns | Low | Low | Some concerns |
| Lv et al. (2019) ^[37]^ | Low | Low | Low | Low | Low | Low | Low |
| Asemi et al. (2013) ^[28]^ | Low | Low | Low | Low | Low | Low | Low |
| Ma et al. (2014) ^[33]^ | Some concerns | Some concerns | Some concerns | Some concerns | Low | Low | Some concerns |
| Moreno-Castilla et al. (2013) ^[29]^ | Low | Low | Low | Low | Low | Low | Low |
| Asemi et al. (2014) ^[30]^ | Low | Low | Low | Low | Low | Low | Low |
| Hernandez et al. (2016) ^[34]^ | Some concerns | Some concerns | Low | Low | Low | Low | Some concerns |
| Xiaoqiong et al. (2024) ^[48]^ | Some concerns | Some concerns | Low | Low | Low | Low | Some concerns |
| Linna et al. (2020) ^[39]^ | Low | Some concerns | Low | Some concerns | Low | Low | Some concerns |
| Hongru et al. (2020) ^[38]^ | Low | Low | Low | Low | Low | Low | Low |
| Zhuqing et al. (2024) ^[51]^ | Low | Low | Low | Low | Low | Low | Low |
| Fengying et al. (2019) ^[36]^ | Some concerns | Some concerns | Some concerns | Some concerns | Low | Low | Some concerns |
| Liyu et al. (2022) ^[45]^ | Low | Low | Low | Low | Low | Low | Low |
| Ke et al. (2022) ^[44]^ | Low | Low | Low | Low | Low | Low | Low |
| Ying et al. (2023) ^[49]^ | Some concerns | Some concerns | Low | Low | Low | Low | Some concerns |
| Bo et al. (2017) ^[35]^ | Low | High | Low | High | Low | Low | High |
| Lixia et al. (2022) ^[46]^ | Low | Some concerns | Low | Low | Low | Some concerns | Some concerns |
| Yuman et al. (2023) ^[50]^ | Low | Low | Low | Low | Low | Low | Low |
| Wen et al. (2022) ^[47]^ | Low | Low | Low | Low | Low | Low | Low |
| Yan et al. (2021) ^[42]^ | Low | Low | Low | Low | Low | Low | Low |

| **FBG for pregnant** | | | | | | |
| --- | --- | --- | --- | --- | --- | --- |
| **Patient or population:** patients with pregnant **Settings:**  **Intervention:** FBG | | | | | | |
| **Outcomes** | **Illustrative comparative risks* (95% CI)** | | **Relative effect (95% CI)** | **No of Participants (studies)** | **Quality of the evidence (GRADE)** | **Comments** |
|  | Assumed risk | Corresponding risk |  |  |  |  |
|  | **Control** | **FBG** |  |  |  |  |
| **FBG** |  | The mean fbg in the intervention groups was **1.01 standard deviations lower** (1.46 to 0.57 lower) |  | 2441 (26 studies) | ⊕⊕⊝⊝ **low**^1,2^ | SMD -1.01 (-1.46 to -0.57) |
| *The basis for the **assumed risk** (e.g. the median control group risk across studies) is provided in footnotes. The **corresponding risk** (and its 95% confidence interval) is based on the assumed risk in the comparison group and the **relative effect** of the intervention (and its 95% CI).  **CI:** Confidence interval; | | | | | | |
| GRADE Working Group grades of evidence **High quality:** Further research is very unlikely to change our confidence in the estimate of effect.  **Moderate quality:** Further research is likely to have an important impact on our confidence in the estimate of effect and may change the estimate. **Low quality:** Further research is very likely to have an important impact on our confidence in the estimate of effect and is likely to change the estimate. **Very low quality:** We are very uncertain about the estimate. | | | | | | |
| ^1^ Some studies had unclear randomization methods. ^2^ I²=96% | | | | | | |

Figure 3.1 GRADE Assessment Results for Fasting blood glucose level

| **2h-PBG for pregnant** | | | | | | |
| --- | --- | --- | --- | --- | --- | --- |
| **Patient or population:** patients with pregnant **Settings:**  **Intervention:** 2h-PBG | | | | | | |
| **Outcomes** | **Illustrative comparative risks* (95% CI)** | | **Relative effect (95% CI)** | **No of Participants (studies)** | **Quality of the evidence (GRADE)** | **Comments** |
|  | Assumed risk | Corresponding risk |  |  |  |  |
|  | **Control** | **2h-PBG** |  |  |  |  |
| **2h-PBG** |  | The mean 2h-pbg in the intervention groups was **0.83 standard deviations lower** (1.08 to 0.57 lower) |  | 2322 (21 studies) | ⊕⊝⊝⊝ **very low**^1,2,3^ | SMD -0.83 (-1.08 to -0.57) |
| *The basis for the **assumed risk** (e.g. the median control group risk across studies) is provided in footnotes. The **corresponding risk** (and its 95% confidence interval) is based on the assumed risk in the comparison group and the **relative effect** of the intervention (and its 95% CI). **CI:** Confidence interval; | | | | | | |
| GRADE Working Group grades of evidence **High quality:** Further research is very unlikely to change our confidence in the estimate of effect.  **Moderate quality:** Further research is likely to have an important impact on our confidence in the estimate of effect and may change the estimate. **Low quality:** Further research is very likely to have an important impact on our confidence in the estimate of effect and is likely to change the estimate. **Very low quality:** We are very uncertain about the estimate. | | | | | | |
| ^1^ Some studies had unclear randomization methods. ^2^ I²=87% ^3^ Egger test=0.019 | | | | | | |

Figure 3.2 GRADE Assessment Results for 2-hour postprandial blood glucose level

| **HOMA-IR for pregnant** | | | | | | |
| --- | --- | --- | --- | --- | --- | --- |
| **Patient or population:** patients with pregnant **Settings:**  **Intervention:** HOMA-IR | | | | | | |
| **Outcomes** | **Illustrative comparative risks* (95% CI)** | | **Relative effect (95% CI)** | **No of Participants (studies)** | **Quality of the evidence (GRADE)** | **Comments** |
|  | Assumed risk | Corresponding risk |  |  |  |  |
|  | **Control** | **HOMA-IR** |  |  |  |  |
| **HOMA-IR** |  | The mean homa-ir in the intervention groups was **0.56 lower** (0.89 to 0.24 lower) |  | 1212 (10 studies) | ⊕⊕⊕⊝ **moderate**^1^ |  |
| *The basis for the **assumed risk** (e.g. the median control group risk across studies) is provided in footnotes. The **corresponding risk** (and its 95% confidence interval) is based on the assumed risk in the comparison group and the **relative effect** of the intervention (and its 95% CI).  **CI:** Confidence interval; | | | | | | |
| GRADE Working Group grades of evidence **High quality:** Further research is very unlikely to change our confidence in the estimate of effect.  **Moderate quality:** Further research is likely to have an important impact on our confidence in the estimate of effect and may change the estimate. **Low quality:** Further research is very likely to have an important impact on our confidence in the estimate of effect and is likely to change the estimate. **Very low quality:** We are very uncertain about the estimate. | | | | | | |
| ^1^ I²=94% | | | | | | |

Figure 3.3 GRADE Assessment Results for Insulin resistance index

| **Cesarean Section for pregnant** | | | | | | |
| --- | --- | --- | --- | --- | --- | --- |
| **Patient or population:** patients with pregnant **Settings:**  **Intervention:** Cesarean Section | | | | | | |
| **Outcomes** | **Illustrative comparative risks* (95% CI)** | | **Relative effect (95% CI)** | **No of Participants (studies)** | **Quality of the evidence (GRADE)** | **Comments** |
|  | Assumed risk | Corresponding risk |  |  |  |  |
|  | **Control** | **Cesarean Section** |  |  |  |  |
| **Cesarean Section** | **Study population** | | **RR 0.54**  (0.28 to 1.03) | 716 (10 studies) | ⊕⊕⊝⊝ **low**^1^ |  |
|  | **296 per 1000** | **160 per 1000** (83 to 305) |  |  |  |  |
|  | **Moderate** | |  |  |  |  |
|  | **230 per 1000** | **124 per 1000** (64 to 237) |  |  |  |  |
| *The basis for the **assumed risk** (e.g. the median control group risk across studies) is provided in footnotes. The **corresponding risk** (and its 95% confidence interval) is based on the assumed risk in the comparison group and the **relative effect** of the intervention (and its 95% CI). **CI:** Confidence interval; **RR:** Risk ratio; | | | | | | |
| GRADE Working Group grades of evidence **High quality:** Further research is very unlikely to change our confidence in the estimate of effect.  **Moderate quality:** Further research is likely to have an important impact on our confidence in the estimate of effect and may change the estimate. **Low quality:** Further research is very likely to have an important impact on our confidence in the estimate of effect and is likely to change the estimate. **Very low quality:** We are very uncertain about the estimate. | | | | | | |
| ^1^ I²=82% | | | | | | |

Figure 3.4 GRADE Assessment Results for Cesarean section rate

| **Macrosomia for pregnant** | | | | | | |
| --- | --- | --- | --- | --- | --- | --- |
| **Patient or population:** patients with pregnant **Settings:**  **Intervention:** Macrosomia | | | | | | |
| **Outcomes** | **Illustrative comparative risks* (95% CI)** | | **Relative effect (95% CI)** | **No of Participants (studies)** | **Quality of the evidence (GRADE)** | **Comments** |
|  | Assumed risk | Corresponding risk |  |  |  |  |
|  | **Control** | **Macrosomia** |  |  |  |  |
| **Macrosomia** | **Study population** | | **RR 0.21**  (0.11 to 0.38) | 929 (11 studies) | ⊕⊕⊕⊝ **moderate**^1^ |  |
|  | **111 per 1000** | **23 per 1000** (12 to 42) |  |  |  |  |
|  | **Moderate** | |  |  |  |  |
|  | **83 per 1000** | **17 per 1000** (9 to 32) |  |  |  |  |
| *The basis for the **assumed risk** (e.g. the median control group risk across studies) is provided in footnotes. The **corresponding risk** (and its 95% confidence interval) is based on the assumed risk in the comparison group and the **relative effect** of the intervention (and its 95% CI). **CI:** Confidence interval; **RR:** Risk ratio; | | | | | | |
| GRADE Working Group grades of evidence **High quality:** Further research is very unlikely to change our confidence in the estimate of effect.  **Moderate quality:** Further research is likely to have an important impact on our confidence in the estimate of effect and may change the estimate. **Low quality:** Further research is very likely to have an important impact on our confidence in the estimate of effect and is likely to change the estimate. **Very low quality:** We are very uncertain about the estimate. | | | | | | |
| ^1^ Some studies had unclear randomization methods. | | | | | | |

Figure 3.5 GRADE Assessment Results for macrosomia

| **Gestational Hypertension for pregnant** | | | | | | |
| --- | --- | --- | --- | --- | --- | --- |
| **Patient or population:** patients with pregnant **Settings:**  **Intervention:** Gestational Hypertension | | | | | | |
| **Outcomes** | **Illustrative comparative risks* (95% CI)** | | **Relative effect (95% CI)** | **No of Participants (studies)** | **Quality of the evidence (GRADE)** | **Comments** |
|  | Assumed risk | Corresponding risk |  |  |  |  |
|  | **Control** | **Gestational Hypertension** |  |  |  |  |
| **Gestational Hypertension** | **Study population** | | **RR 0.25**  (0.11 to 0.57) | 780 (8 studies) | ⊕⊕⊕⊝ **moderate**^1^ |  |
|  | **64 per 1000** | **16 per 1000** (7 to 37) |  |  |  |  |
|  | **Moderate** | |  |  |  |  |
|  | **64 per 1000** | **16 per 1000** (7 to 36) |  |  |  |  |
| *The basis for the **assumed risk** (e.g. the median control group risk across studies) is provided in footnotes. The **corresponding risk** (and its 95% confidence interval) is based on the assumed risk in the comparison group and the **relative effect** of the intervention (and its 95% CI). **CI:** Confidence interval; **RR:** Risk ratio; | | | | | | |
| GRADE Working Group grades of evidence **High quality:** Further research is very unlikely to change our confidence in the estimate of effect.  **Moderate quality:** Further research is likely to have an important impact on our confidence in the estimate of effect and may change the estimate. **Low quality:** Further research is very likely to have an important impact on our confidence in the estimate of effect and is likely to change the estimate. **Very low quality:** We are very uncertain about the estimate. | | | | | | |
| ^1^ Some studies had unclear randomization methods. | | | | | | |

Figure 3.6 GRADE Assessment Results for Preeclampsia

| **Preterm Birth for pregnant** | | | | | | |
| --- | --- | --- | --- | --- | --- | --- |
| **Patient or population:** patients with pregnant **Settings:**  **Intervention:** Preterm Birth | | | | | | |
| **Outcomes** | **Illustrative comparative risks* (95% CI)** | | **Relative effect (95% CI)** | **No of Participants (studies)** | **Quality of the evidence (GRADE)** | **Comments** |
|  | Assumed risk | Corresponding risk |  |  |  |  |
|  | **Control** | **Preterm Birth** |  |  |  |  |
| **Preterm Birth** | **Study population** | | **RR 0.49**  (0.32 to 0.74) | 1071 (6 studies) | ⊕⊕⊕⊕ **high** |  |
|  | **110 per 1000** | **54 per 1000** (35 to 81) |  |  |  |  |
|  | **Moderate** | |  |  |  |  |
|  | **57 per 1000** | **28 per 1000** (18 to 42) |  |  |  |  |
| *The basis for the **assumed risk** (e.g. the median control group risk across studies) is provided in footnotes. The **corresponding risk** (and its 95% confidence interval) is based on the assumed risk in the comparison group and the **relative effect** of the intervention (and its 95% CI). **CI:** Confidence interval; **RR:** Risk ratio; | | | | | | |
| GRADE Working Group grades of evidence **High quality:** Further research is very unlikely to change our confidence in the estimate of effect.  **Moderate quality:** Further research is likely to have an important impact on our confidence in the estimate of effect and may change the estimate. **Low quality:** Further research is very likely to have an important impact on our confidence in the estimate of effect and is likely to change the estimate. **Very low quality:** We are very uncertain about the estimate. | | | | | | |

Figure 3.7 GRADE Assessment Results for Preterm Birth

# Supplementary 4: Publication bias


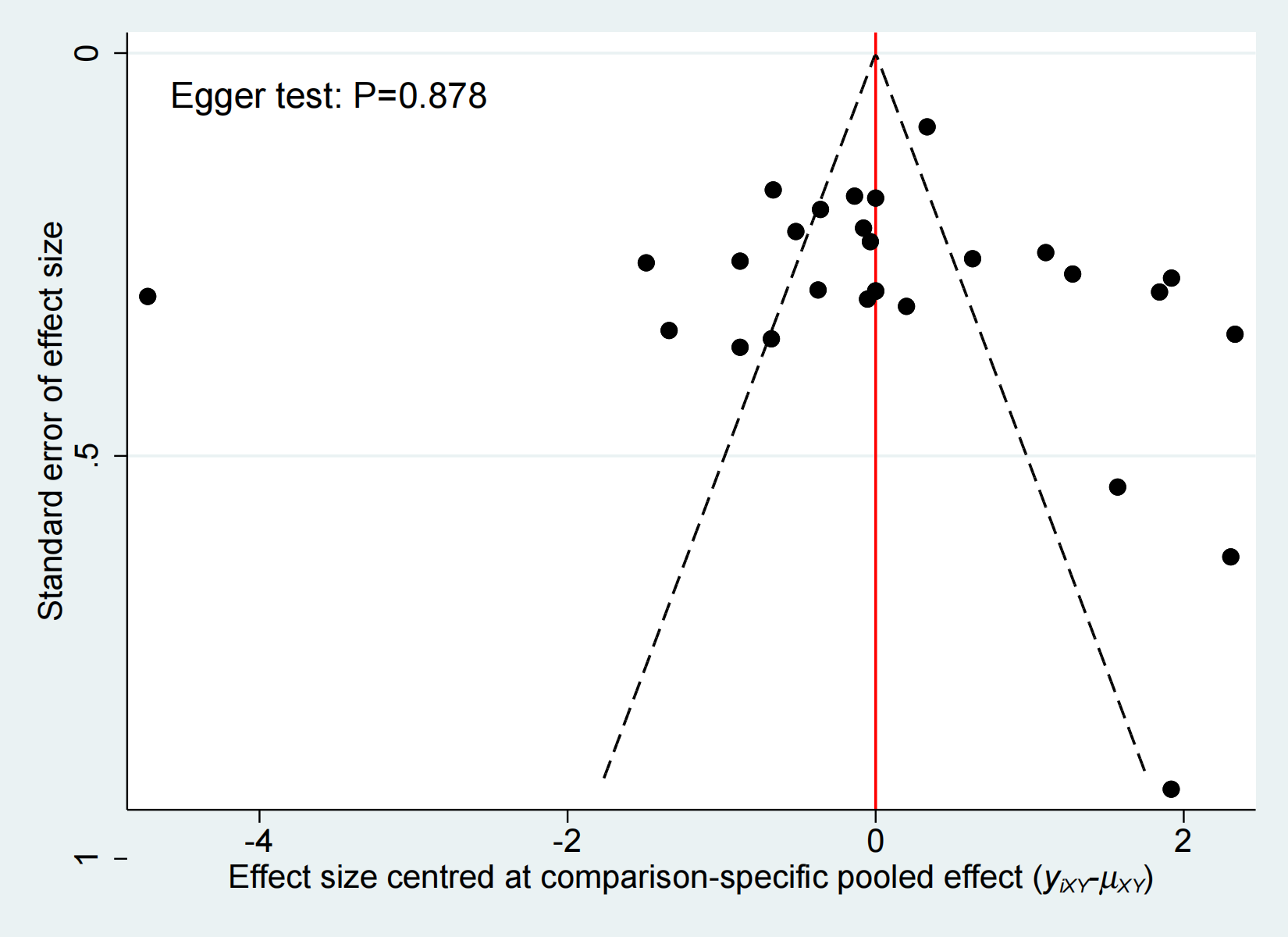


Figure 4.1 The funnel plot of Fasting blood glucose level. The result of Egger test showed the p=0.878.


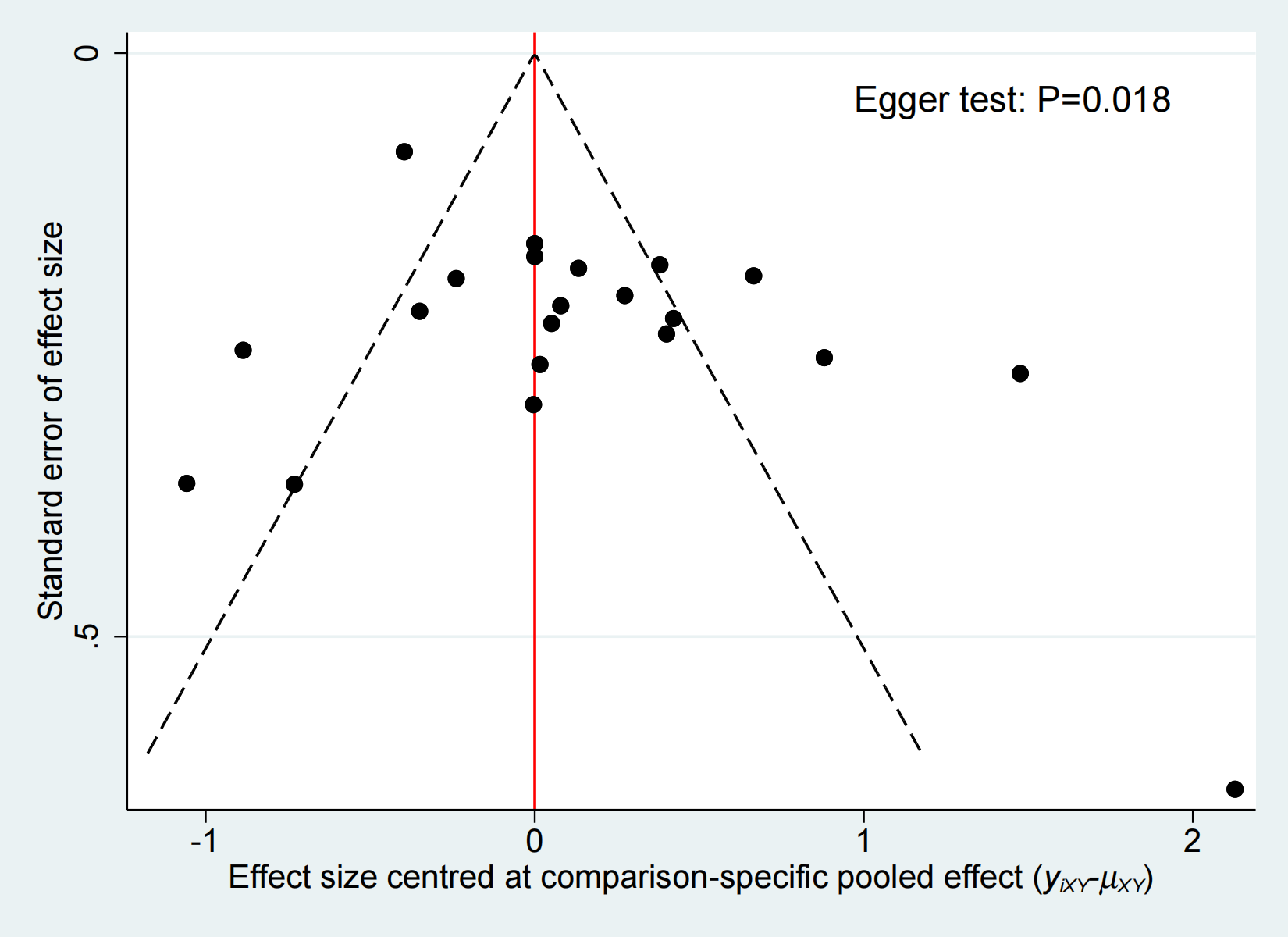


Figure 4.2 The funnel plot of 2-hour postprandial blood glucose level. The result of Egger test showed the p=0.018.


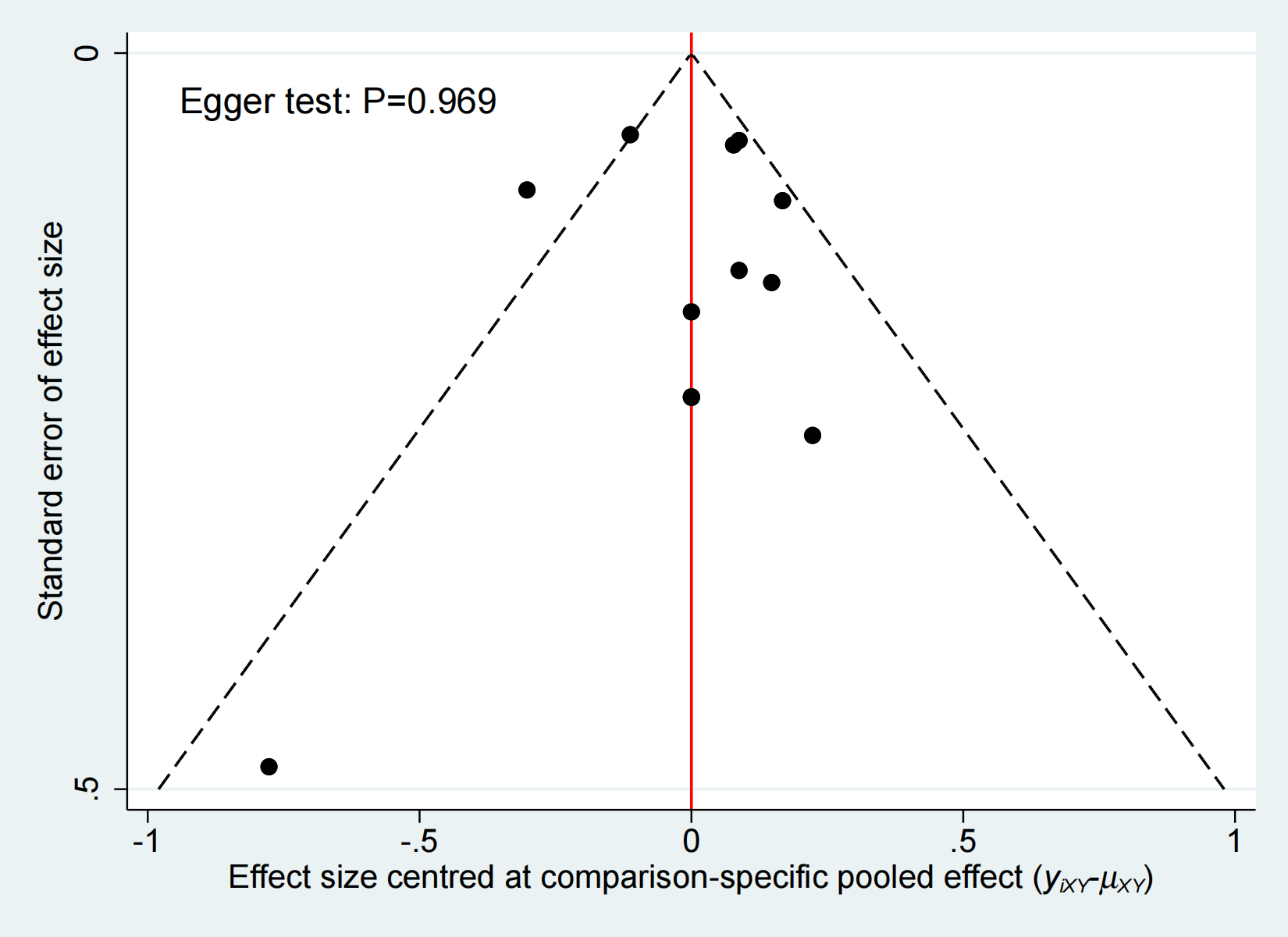


Figure 4.3 The funnel plot of Insulin resistance index. The result of Egger test showed the p=0.969.


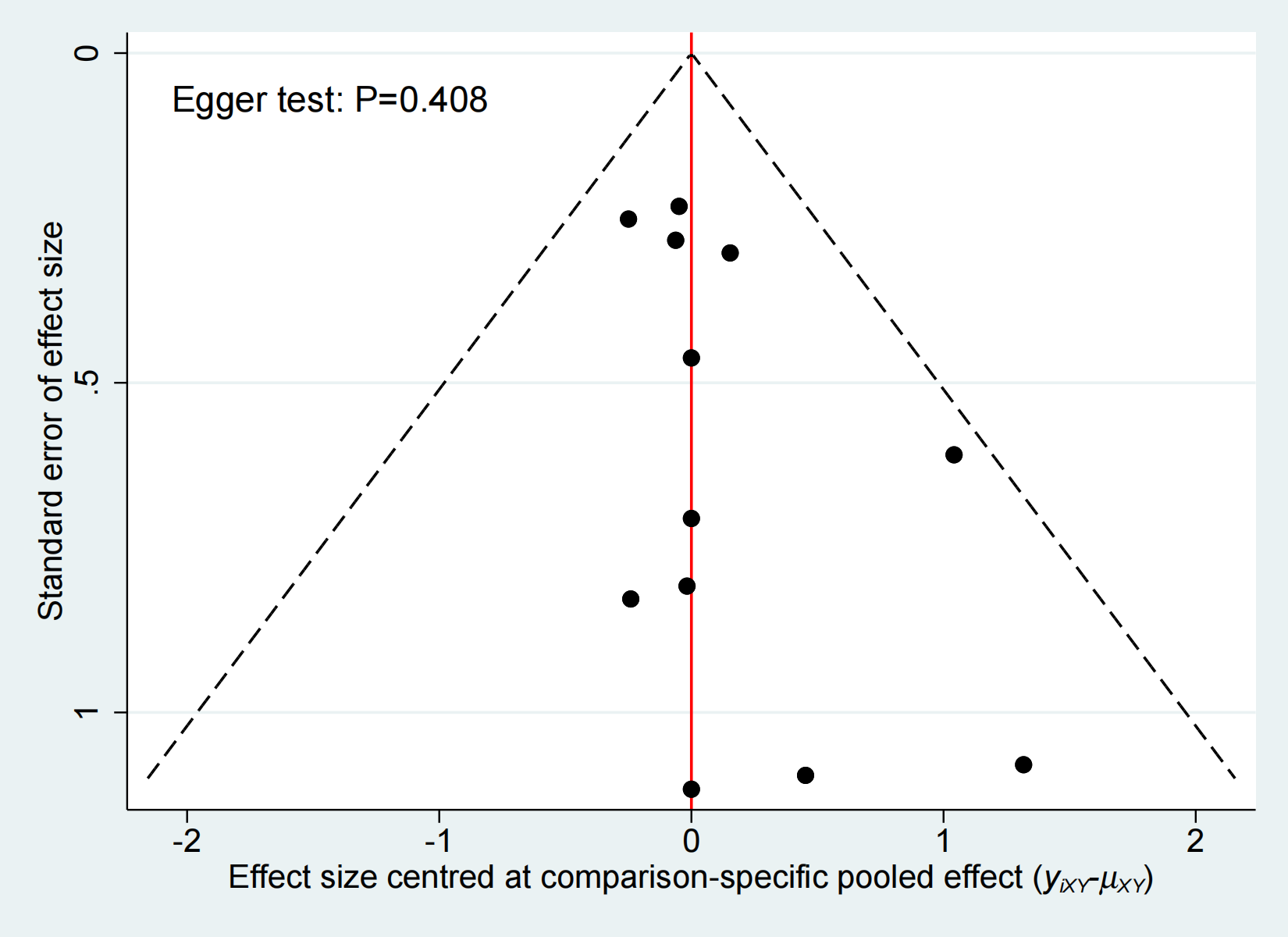


Figure 4.4 The funnel plot of Cesarean section rate. The result of Egger test showed the p=0.408.


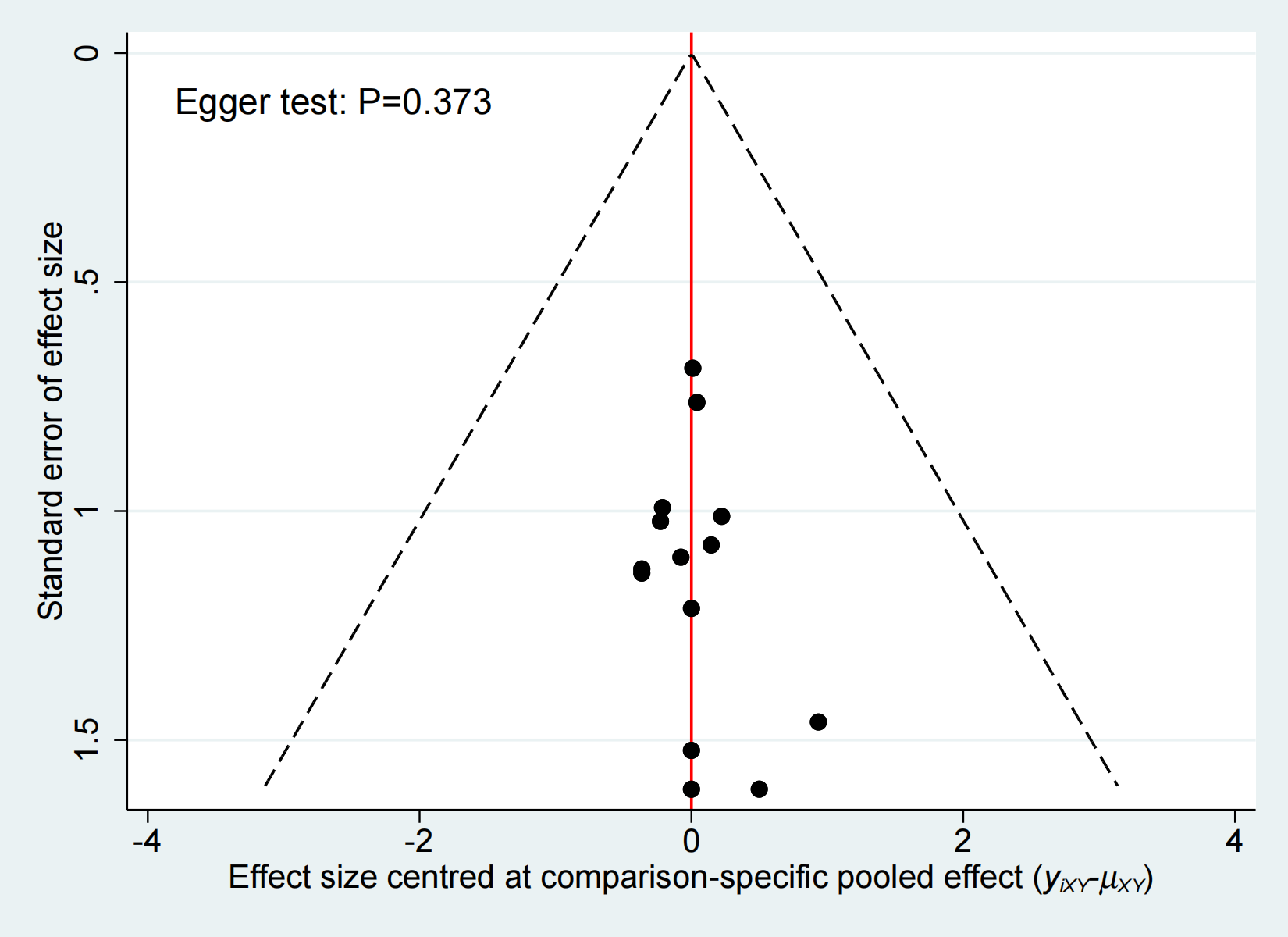


Figure 4.5 The funnel plot of Incidence of macrosomia. The result of Egger test showed the p=0.373.


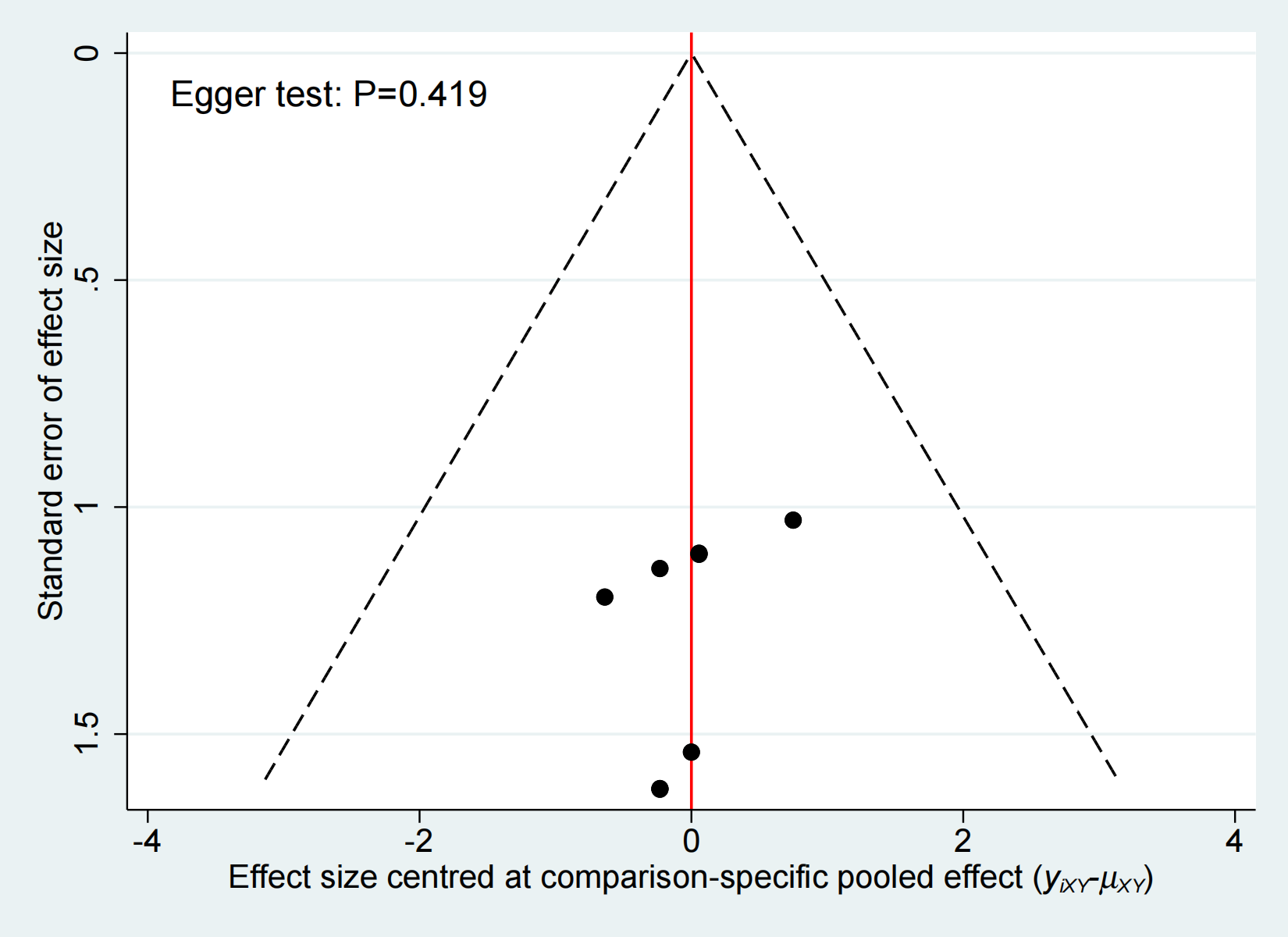


Figure 4.6 The funnel plot of Gestational Hypertension. The result of Egger test showed the p=0.419.


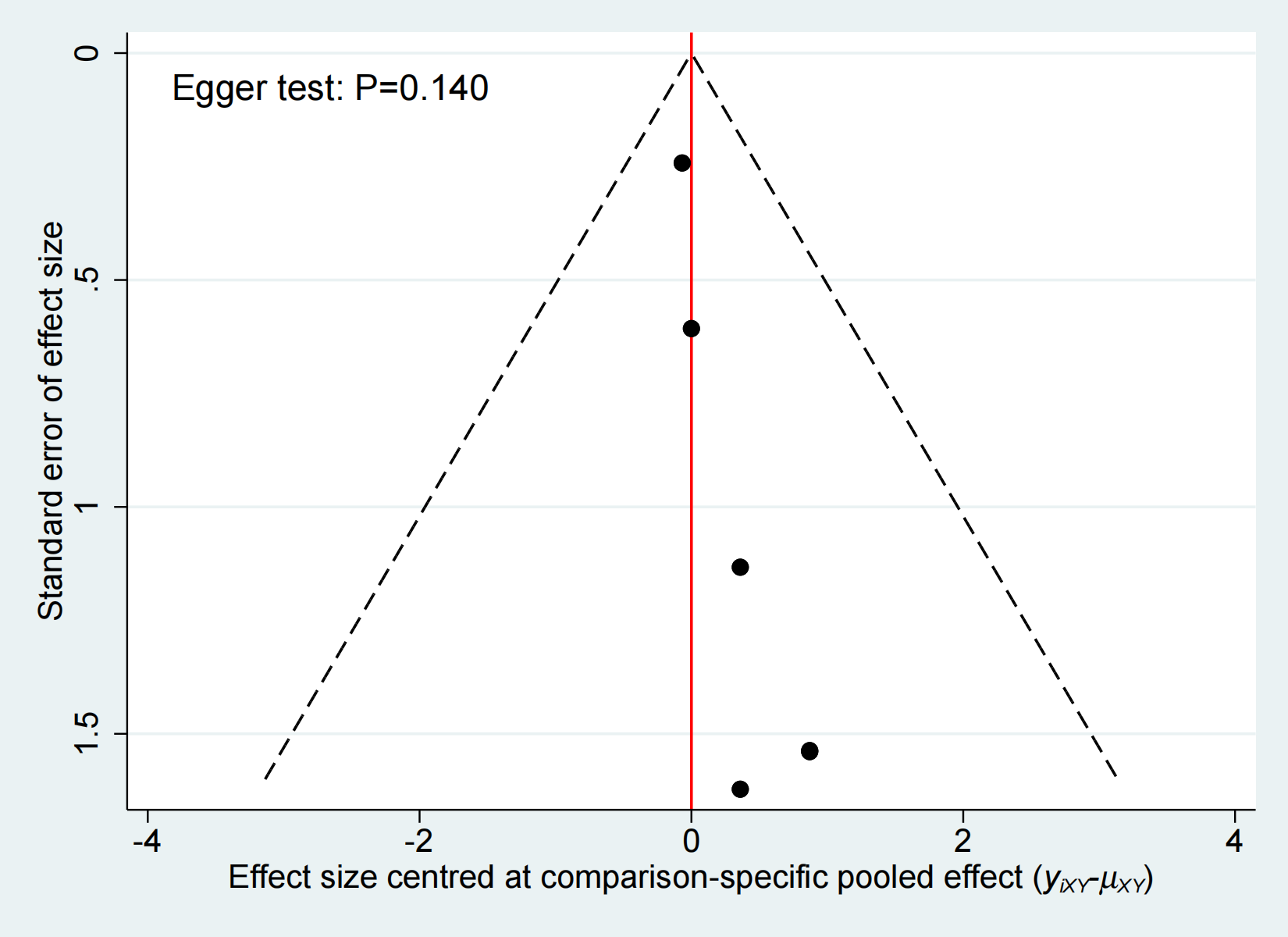


Figure 4.7 The funnel plot of Preterm birth. The result of Egger test showed the p=0.140

# Supplementary 5: League table of outcomes

Table 5.1 FBG

| DASH diet |  |  |  |  |  |
| --- | --- | --- | --- | --- | --- |
| -1.10 (-3.66,1.47) | Low-carb diet |  |  |  |  |
| -1.39 (-4.70,1.92) | -0.29 (-3.06,2.48) | Low-carb DASH diet |  |  |  |
| -1.52 (-3.46,0.41) | -0.42 (-2.37,1.52) | -0.13 (-2.99,2.72) | Low-GI diet |  |  |
| -1.54 (-5.03,1.96) | -0.44 (-3.94,3.06) | -0.15 (-4.22,3.93) | -0.01 (-3.08,3.05) | Low-GL diet |  |
| **-2.35 (-4.15,-0.54)** | -1.25 (-3.07,0.57) | -0.96 (-3.73,1.81) | -0.82 (-1.52,-0.13) | -0.81 (-3.80,2.18) | Standard diet |

Table 5.2 2h-PBG

| DASH diet |  |  |  |  |
| --- | --- | --- | --- | --- |
| **-1.41 (-2.56,-0.25)** | Low-GI diet |  |  |  |
| **-1.84 (-3.52,-0.16)** | -0.43 (-1.74,0.87) | Low-GL diet |  |  |
| **-2.21 (-3.89,-0.53)** | -0.81 (-2.11,0.49) | -0.37 (-2.16,1.41) | Low-carb diet |  |
| **-2.21 (-3.32,-1.10)** | **-0.81 (-1.12,-0.49)** | -0.37 (-1.64,0.89) | -0.00 (-1.26,1.26) | Standard diet |

Table 5.3 HOMA-IR

| DASH diet |  |  |  |  |
| --- | --- | --- | --- | --- |
| **-1.23 (-1.78,-0.67)** | Low-GI diet |  |  |  |
| **-1.37 (-2.12,-0.63)** | -0.15 (-0.67,0.38) | Low-carb DASH diet |  |  |
| **-1.76 (-2.50,-1.02)** | **-0.53 (-1.06,-0.01)** | -0.38 (-0.82,0.05) | Low-carb diet |  |
| **-1.90 (-2.44,-1.36)** | **-0.67 (-0.80,-0.54)** | **-0.53 (-1.03,-0.02)** | -0.14 (-0.65,0.36) | Standard diet |

Table 5.4 Cesarean Section

| Low-GI diet |  |  |  |  |  |
| --- | --- | --- | --- | --- | --- |
| 0.85 (0.22,3.29) | Low-GL diet |  |  |  |  |
| 0.72 (0.25,2.06) | 0.85 (0.33,2.20) | DASH diet |  |  |  |
| 0.40 (0.07,2.10) | 0.47 (0.09,2.34) | 0.55 (0.14,2.16) | Low-carb DASH diet |  |  |
| 0.40 (0.13,1.21) | 0.47 (0.17,1.30) | **0.56 (0.33,0.96)** | 1.02 (0.26,4.04) | Low-carb diet |  |
| 0.39 (0.14,1.07) | 0.46 (0.19,1.14) | **0.54 (0.40,0.74)** | 0.99 (0.26,3.76) | 0.97 (0.62,1.52) | Standard diet |

Table 5.5 Macrosomia

| DASH diet |  |  |  |  |  |
| --- | --- | --- | --- | --- | --- |
| 0.54 (0.10,2.79) | Low-GI diet |  |  |  |  |
| 0.65 (0.03,16.64) | 1.20 (0.06,25.14) | Low-carb DASH diet |  |  |  |
| 0.25 (0.02,3.96) | 0.46 (0.04,5.76) | 0.39 (0.01,16.68) | Low-GL diet |  |  |
| 0.25 (0.04,1.43) | 0.46 (0.12,1.75) | 0.38 (0.02,7.46) | 0.99 (0.07,13.27) | Low-carb diet |  |
| **0.12 (0.03,0.51)** | **0.23 (0.10,0.54)** | 0.19 (0.01,3.59) | 0.50 (0.05,5.38) | 0.51 (0.18,1.44) | Standard diet |

Table 5.6 Gestational Hypertension

| Low-GL diet |  |  |
| --- | --- | --- |
| 0.76 (0.03,17.60) | Low-GI diet |  |
| 0.20 (0.01,4.09) | **0.26 (0.11,0.65)** | Standard diet |

Table 5.7 Preterm Birth

| Low-GI diet |  |  |
| --- | --- | --- |
| 0.70 (0.20,2.49) | Low-GL diet |  |
| **0.48 (0.30,0.75)** | 0.68 (0.21,2.24) | Standard diet |

Note: Boldface indicates significant difference.
